# The Efficacy of Dietary Intake, Supplementation, and Blood Concentrations of Carotenoids in Cancer Prevention: Insights from an Umbrella Meta-Analysis

**DOI:** 10.3390/foods13091321

**Published:** 2024-04-25

**Authors:** Jing Sui, Jingwen Guo, Da Pan, Ying Wang, Ying Xu, Guiju Sun, Hui Xia

**Affiliations:** 1 Research Institute for Environment and Health, Nanjing University of Information Science and Technology, Nanjing 210044, China; suijingseu@163.com (J.S.); szyyyjgl@163.com (J.G.); xuyingzoe@163.com (Y.X.); 2 Key Laboratory of Environmental Medicine Engineering, Ministry of Education, School of Public Health, Southeast University, Nanjing 210009, China; pantianqi92@foxmail.com (D.P.); yingwang04@126.com (Y.W.); gjsun@seu.edu.cn (G.S.)

**Keywords:** carotenoids, cancer, supplement risk, meta-analysis, umbrella review

## Abstract

Previous meta-analyses of multiple studies have suggested that dietary intake and blood concentrations of carotenoids, as well as dietary supplement of certain carotenoids, play a role in reducing the risk of cancer. However, the conclusions of these studies have been subject to controversy. We conducted an umbrella review of meta-analyses to comprehensively analyze and evaluate the evidence pertaining the association between carotenoids and cancer outcomes. We searched PubMed, Web of Science, Embase, and Cochrane Library databases of meta-analyses and systematic reviews up to June 2023. Our selection criteria encompassed meta-analyses of cohort and case-control studies, as well as randomized controlled clinical trials, which investigated the associations between carotenoids and cancer risk. We also determined the levels of evidence for these associations with AMSTAR 2 criteria. We included 51 eligible articles, including 198 meta-analyses for qualitative synthesis in the umbrella review. Despite the presence of moderate to high heterogeneity among the studies, dietary intake, supplementation, and blood concentrations of carotenoids were inversely associated with the risk of total cancer, and certain specific cancers of lung, digestive system, prostate, breast, head and neck, and others. Subgroup analysis also showed that individual carotenoids (α-carotene, β-carotene, β-cryptoxanthin, lutein, zeaxanthin, and lycopene) offer certain protection against specific types of cancers. However, high doses of carotenoid supplements, especially β-carotene, significantly increased the risk of total cancer, lung cancer, and bladder cancer. Our umbrella meta-analysis supported that high intake of dietary carotenoids as a whole food approach could be more beneficial in reducing cancer risk. Concurrently, the findings suggest that the efficacy of single-carotenoid supplementation in cancer prevention remains a subject of controversy.

## 1. Introduction

The precise pathogenic mechanisms underlying carcinogenesis remain elusive, but current theories suggest that it is a multistep process characterized by the accumulation of cellular injuries at various biological levels, including genetic and epigenetic changes [1]. Diet and dietary supplements are widely recognized as potential inhibitors of carcinogenic process [2]. Accumulating evidence from epidemiologic studies demonstrates that high consumption of fruits and vegetables is protective against numerous types of cancer [3,4]. Carotenoids, natural fat-soluble pigments found abundantly in yellow, orange, and red fruits and vegetables (such as oranges, tomatoes, and carrots), constitute an important part of the human diet with intense antioxidant properties [5,6]. Since the human body does not synthesize carotenoids, they must be obtained from dietary sources or supplements. Carotenoids are categorized into two groups: hydrocarbons, such as α-carotene, β-carotene, and lycopene, and xanthophylls, such as β-cryptoxanthin, lutein, zeaxanthin, and lycopene [7]. Multiple carotenoids, such as α-carotene, β-carotene, β-cryptoxanthin, lutein, zeaxanthin, and lycopene, which are acquired through diet, can be examined in plasma and tissues [8]. Numerous epidemiological studies have found that a higher dietary consumption of carotenoids is associated with a lower risk of several chronic diseases [9,10].

Carotenoids have been shown to possess antioxidant potential and immunoenhancing properties in both in vitro and in vivo studies. These compounds can reduce chromosome aberrations, inhibit the formation of malignant tumors, decrease DNA damage, regulate gap-junction communication between cells, and reduce cell proliferation and transformation [11]. However, the precise contribution of dietary carotenoids or serum carotenoids to the risk of various cancer types remains a subject of controversy due to inconsistent findings from epidemiologic studies. Furthermore, it is important to note that the current meta-analysis focuses on published studies that presented their results primarily through randomized/fixed-effect sizes, 95% CIs, and *p*-values, which were susceptible to small-study effects and heterogeneity [12]. Therefore, there is a need for a systematic and comprehensive approach to provide a clearer understanding of the relationship between carotenoids and cancer risk.

The growing number of meta-analyses in the field of human health outcomes does not always translate into improved medical guidance, as these studies often come with certain limitations. Recognizing these limitations, Ioannidis et al. [13] first introduced the concept of umbrella reviews back in 2009. Recently, umbrella reviews have provided systematic computation and evaluation of meta-analyses and have been widely used to assess associations between various factors (nutrition, risk factors, behaviors) and human health outcomes, including mortality, cardiovascular disease, type 2 diabetes mellitus, and multiple cancers, thereby improving the accuracy and strength of results [14,15,16,17]. To the best of our knowledge, no previous umbrella reviews of meta-analyses have investigated the association between carotenoids and cancer risk. To further understand and reassess the association, we conducted the first-ever such umbrella review by collecting all available meta-analyses to explore potential strategies for cancer prevention, and enhance the strength and validity of the evidence.

## 2. Materials and Methods

The present umbrella review of meta-analyses was performed in accordance with the guidelines in the Preferred Reporting Items for Systematic Reviews and Meta-Analyses (PRISMA) [18]. We are registered in PROSPERO (Registration No. CRD42023417600).

### 2.1. Literature Search Strategy

We performed an umbrella review of the systematic reviews and meta-analyses on associations between carotenoid consumption and cancer risk. Two investigators (J.S., J.G.) performed the search from PubMed, Web of Science, Embase, and Cochrane Library databases limited to English up to June 2023. The search terms were as follows: “(carotenoids OR α-carotene OR alpha carotene OR beta carotene OR β-carotene OR zeta Carotene OR ζ-carotene OR β-cryptoxanthin OR lutein OR zeaxanthin OR lycopene OR phytoene OR phytofluene OR violaxanthin OR neoxanthin OR astaxanthin) AND (cancer OR tumor OR neoplasm OR neoplasia) AND (systematic review OR meta-analysis)”. The references of all identified articles were also manually viewed.

### 2.2. Eligibility and Inclusion/Exclusion Criteria

Systematic reviews or meta-analyses assessing associations between carotenoid consumption and cancer risk were included. The inclusion criteria were as follows: (i) meta-analyses of cohort and case-control studies and randomized controlled trials (RCTs) investigating the effect of dietary, blood, and supplement of carotenoids on the cancer risk; (ii) considering the incidence or mortality of cancer as the outcome; (iii) reporting the effect sizes (OR, odds ratio; RR, relative risk; HR, hazard ratio) and corresponding confidence intervals (CIs); (iv) published in English.

The exclusion criteria were as follows: (i) meta-analyses of non-observational studies or non-RCT; (ii) without original data to analyze the summary risk estimate, 95% CIs; (iii) systematic reviews without meta-analysis; (iv) articles, letters, editorials, and conference abstracts; (v) duplicated publications.

**Figure 1:**
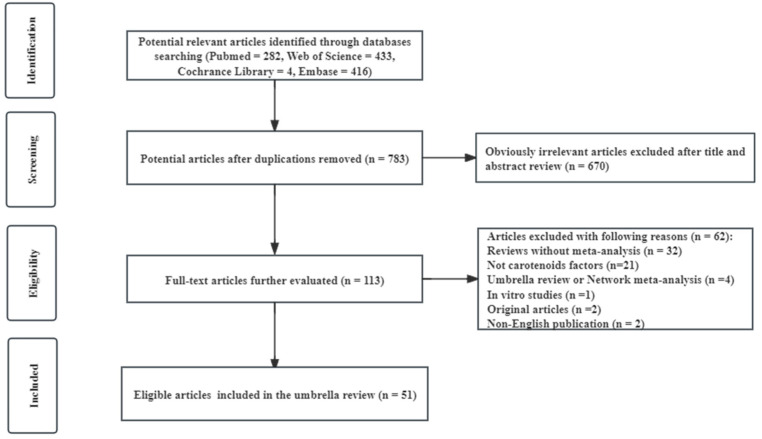
Flow chart of the literature search.

A detailed flow chart of the screening and selection process of eligible articles is presented in Figure 1.

### 2.3. Ata Extraction and Quality Assessment

Two investigators (Y.W. and Y.X.) independently extracted the following information from each eligible paper: the first author’s name, publication year, type of cancer outcomes, type of carotenoids, study design (cohort, case control, RCTs), number of cases/control or total participants, meta-analysis metric, OR/RR/HR and CIs, number of included studies in meta-analysis, effect model, and assessment tool of the original study.

A MeaSurement Tool to Assess systematic Reviews 2 (AMSTAR 2) was used to evaluate the methodological quality of eligible meta-analyses [19]. A total of 16 items, including 7 critical and 9 non-critical domains, constituted the AMSTAR 2. According to the quality of each item, we further scored each eligible meta-analysis into High, Moderate, Low, or Critical low quality.

### 2.4. Data Analysis

In this umbrella review, we extracted OR/RR/HR and 95% CI data from each eligible meta-analysis to re-analyze the association between consumption of carotenoids and cancer risk. I^2^ and Cochran Q tests were used to assess the heterogeneity between included studies [20]. I^2^ > 50% and *p* value < 0.10 indicated significant heterogeneity and calculated with the random-effects model; otherwise, the fixed-effects model was performed. Publication bias and the small-study effect were assessed by the Egger test and funnel plot [21]. For heterogeneity and publication bias, a *p* value < 0.05 was adopted as a significance threshold as the result of the small-study effects. For other tests, a significance threshold at the level of *p* value < 0.05 was considered. Moreover, subgroup evaluation was carried out by the type of carotenoids, such as α-carotene, β-carotene, ζ-carotene, and lycopene. All statistical analyses were evaluated with Comprehensive Meta Analysis (CMA) version 3.3.

## 3. Results

### 3.1. Study Identification

A total of 1135 articles were initially identified from four databases (PubMed, Web of Science, Cochrane Library, and Embase databases), and 51 eligible articles with 198 meta-analyses were included in our review after exclusions (Table 1). All eligible articles were published between 2000 and 2023. Our study aimed to systematically categorize 198 meta-analyses into eight distinct categories of cancer risk. These categories included total cancer, lung cancer, digestive system cancer, prostate cancer, breast cancer, bladder cancer, head and neck cancer, and gynecologic/skin/blood cancer [22,23,24,25,26,27,28,29,30,31,32,33,34,35,36,37,38,39,40,41,42,43,44,45,46,47,48,49,50,51,52,53,54,55,56,57,58,59,60,61,62,63,64,65,66,67,68,69,70,71,72,73]. Due to the limited number of meta-analyses available, gynecologic/skin/blood cancer was evaluated as a group.

### 3.2. The Quality Assessment of Included Meta-Analyses

In the terms of quality of included meta-analyses, results from the AMSTAR 2 questionnaire showed that present umbrella meta-analyses included 41 studies assessed as high quality, 19 studies as moderate quality, and 138 studies as low or critically low quality, respectively (Table S1).

### 3.3. Total Cancer Outcomes

A total of 198 effect meta-analyses were reported in all eligible meta-analyses examining the relationship between dietary consumption/supplementation/blood level and cancer outcomes. The studies were on total cancer (*n* = 26) and six other distinct categories of cancer (*n* = 172). Our study has revealed a significant correlation between carotenoids and cancer risk (OR: 0.860; 95% CI: 0.840–0.881; *p* < 0.001) (Supplemental Files, Figure S1) with a random-effect model (I^2^ = 0.766, *p* < 0.001). Regarding subgroup evaluation, we observed that total carotenoids (OR: 0.743; 95% CI: 0.675–0.819), α-carotene (OR: 0.838; 95% CI: 0.797–0.881), β-carotene (OR: 0.906; 95% CI: 0.875–0.938), lutein and zeaxanthin (OR: 0.850; 95% CI: 0.797–0.906), β-cryptoxanthin (OR: 0.785; 95% CI: 0.697–0.883), and lycopene (OR: 0.886; 95% CI: 0.858–0.916) protected against total cancer (Table 2). The assessment of publication bias of funnel plot by Egger’s regression test showed evidence of small-study effect in the present umbrella meta-analysis (*p* < 0.001), while results from trim and fill analysis with 75 imputed studies showed that the overall effect was not significantly confounded by the bias (OR = 0.945; 95% CI: 0.921–0.970).

### 3.4. Lung Cancer Outcomes

**Figure 2:**
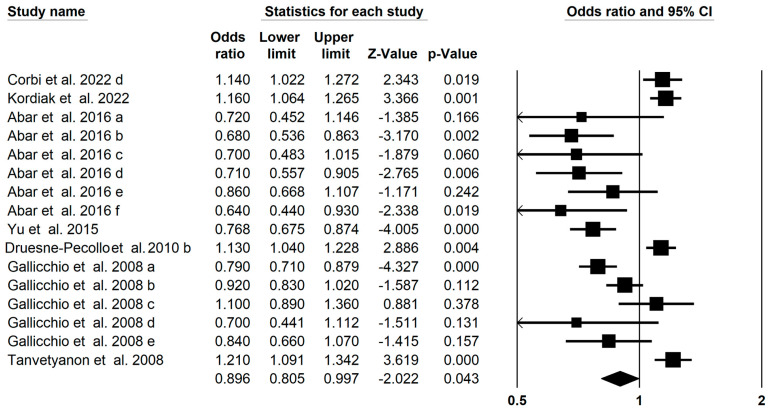
Forest plot of the effect of carotenoids on lung cancer [26,27,49,50,68,69].

Sixteen meta-analyses of the association of carotenoids and lung cancer were identified. The present umbrella meta-analysis demonstrated that carotenoids could significantly reduce the risk of lung cancer (OR = 0.896; 95% CI: 0.805–0.997; *p* = 0.04, Figure 2) with a high heterogeneity (I^2^ = 0.864, *p* < 0.001). Further subgroup analysis showed a significant effect of total carotenoids on the risk of lung cancer (OR: 0.774; 95% CI: 0.700–0.855) (Table 2). Nevertheless, four studies showed that β-carotene intake significantly increased the lung cancer risk (OR = 1.21; 95% CI: 1.09–1.34; OR = 1.13; 95% CI: 1.04–1.23; OR = 1.16; 95% CI: 1.06–1.26; OR = 1.14; 95% CI: 1.02–1.27) [27,30,68,69]. The assessment of publication bias of the funnel plot by Begg regression test showed no publication bias in the present umbrella meta-analysis (*p* = 0.34). Seven imputed studies subjected to trim and fill analysis suggested that there was no statistically significant association between carotenoids and lung cancer risk (OR = 1.033; 95% CI: 0.929–1.147).

### 3.5. Digestive System Cancer Outcomes

**Figure 3:**
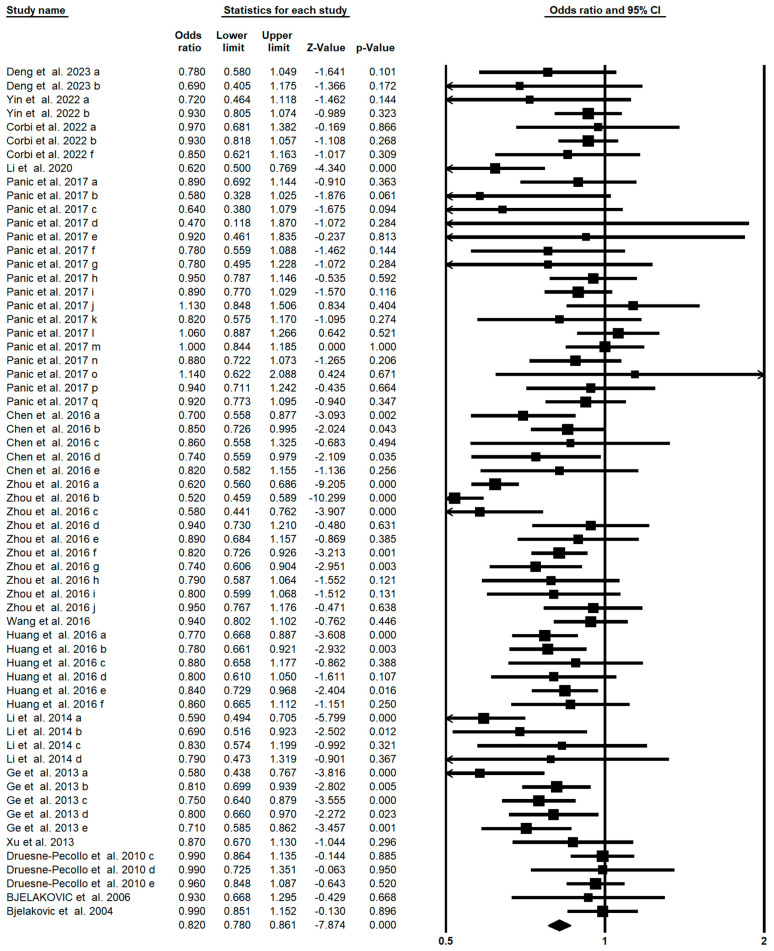
Forest plot of the effect of carotenoids on digestive system cancer [23,25,30,40,41,42,51,52,54,55,58,66,68,70,72].

Among 62 meta-analyses, 18 showed a statistically significant result for reduction of digestive system cancer risk with carotenoids. As shown in Figure 3, higher consumption/blood level of carotenoids resulted in a significant decrease in digestive system cancer (OR = 0.820; 95% CI: 0.780–0.861; *p* < 0.001), which is concluded from a random-effect model since there was a moderate heterogeneity (I^2^ = 0.675, *p* < 0.001). Further subgroup analysis showed a significant effect of total carotenoids (OR: 0.811; 95% CI: 0.674–0.975), α-carotene (OR: 0.792; 95% CI: 0.707–0.887), β-carotene (OR: 0.799; 95% CI: 0.717–0.890), lutein and zeaxanthin (OR: 0.856; 95% CI: 0.794–0.923), β-cryptoxanthin (OR: 0.790; 95% CI: 0.698–0.894), and lycopene (OR: 0.873; 95% CI: 0.825–0.924) on the risk of digestive system cancer (Table 2). We also synthetically analyzed the role of carotenoids in different types of digestive cancers. Our study found a significantly protective effect of carotenoids on the risk of gastric cancer (OR: 0.749; 95% CI: 0.668–0.841), colorectal cancer (OR: 0.932; 95% CI: 0.887–0.979), esophageal cancer (OR: 0.752; 95% CI: 0.671–0.844), and pancreatic cancer (OR: 0.812; 95% CI: 0.765–0.861) (Table 2). The results showed that the assessment of publication bias of the funnel plot by Egger’s regression test showed no publication bias in the umbrella meta-analysis (*p* = 0.77).

### 3.6. Prostate Cancer Outcomes

**Figure 4:**
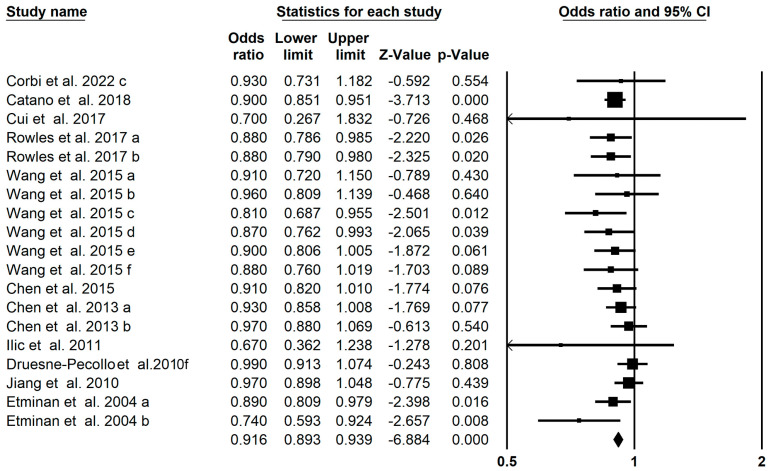
Forest plot of the effect of carotenoids on prostate cancer [24,30,31,32,39,42,48,57,60,63,68].

The pooled effect of carotenoids on prostate cancer was concluded from 19 meta-analyses in 11 studies, which indicated a significant decrease in prostate cancer risk (OR = 0.916; 95% CI: 0.893–0.939; *p* < 0.001, Figure 4), and found insignificant between-study heterogeneity (I^2^ = 0, *p* = 0.514). The subgroup analysis showed that the significant effect of α-carotene (OR: 0.880; 95% CI: 0.784–0.987) and lycopene (OR: 0.899; 95% CI: 0.872–0.927) on the risk of prostate cancer (Table 2). The Egger’s regression test showed no publication bias in the umbrella meta-analysis (*p* = 0.06). While further trim and fill analysis with 5 imputed studies suggested that the impacts of carotenoids on prostate cancer were still significant (OR = 0.923; 95% CI: 0.899–0.949).

### 3.7. Breast Cancer Outcomes

**Figure 5:**
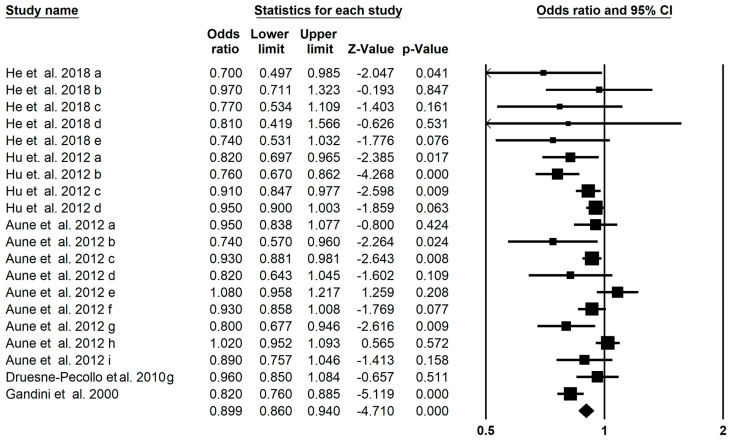
Forest plot of the effect of carotenoids on breast cancer [22,30,35,37,64].

The result of 20 meta-analyses of the association of carotenoids and breast cancer showed total carotenoids could significantly decrease the risk of breast cancer (OR = 0.899; 95% CI: 0.860–0.940; *p* < 0.001, Figure 5) with a significantly moderate heterogeneity (I^2^ = 0.613, *p* < 0.001). Further subgroup analysis showed a significant effect of α-carotene (OR: 0.900; 95% CI: 0.857–0.945), and β-carotene (OR: 0.896; 95% CI: 0.833–0.964) on the risk of breast cancer (Table 2). The assessment of publication bias of the funnel plot by Egger’s regression test showed insignificant publication bias in the umbrella meta-analysis (*p* = 0.053). Six imputed studies subjected to trim and fill analysis suggested that carotenoids were protective against breast cancer (OR = 0.930; 95% CI: 0.888–0.974).

### 3.8. Bladder Cancer Outcomes

**Figure 6:**
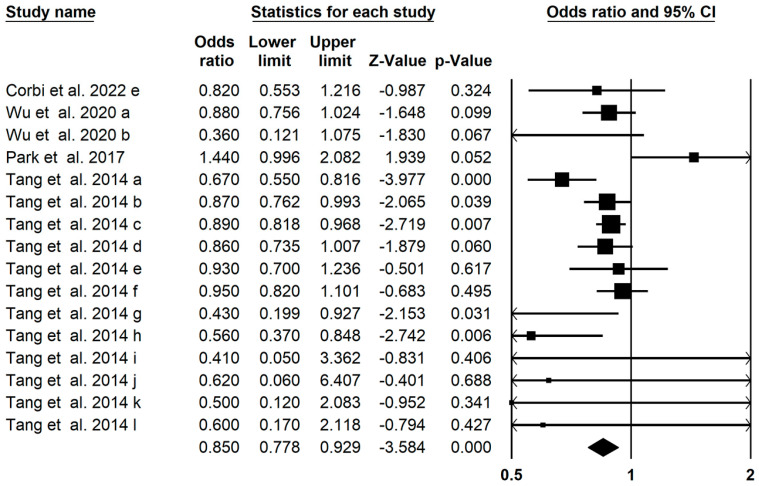
Forest plot of the effect of carotenoids on bladder cancer [44,59,67,68].

The pooled effect of carotenoids on prostate cancer was concluded from 15 meta-analyses in 3 studies, which indicated a significant decrease in prostate cancer risk (OR = 0.850; 95% CI: 0.778–0.929; *p* = 0.001, Figure 6), and found low between-study heterogeneity (I^2^ = 0.489, *p* = 0.017). Further subgroup analysis showed a significant effect of total carotenoids (OR: 0.631; 95% CI: 0.469–0.849) on the risk of bladder cancer (Table 2). The assessment of publication bias of funnel plot by Egger’s regression test showed insignificant publication bias in the umbrella meta-analysis (*p* = 0.108). Five imputed studies subjected to trim and fill analysis suggested that carotenoids were protective against bladder cancer (OR = 0.882; 95% CI: 0.801–0.971).

### 3.9. Head and Neck Cancer Outcomes

**Figure 7:**
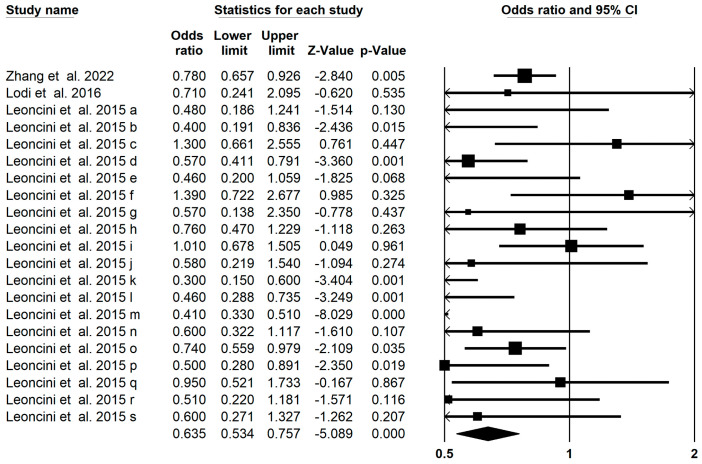
Forest plot of the effect of carotenoids on head and neck cancer [47,53,73].

High-serum or high intake or high-supplement concentration of carotenoids were associated with significant reductions in the risk of head and neck cancer (OR = 0.635; 95% CI: 0.534–0.757; *p* < 0.001, Figure 7) with a moderate heterogeneity (I^2^ = 0.567, *p* < 0.001). In terms of carotenoids, significant decreases were observed in subgroups of patients with head and neck cancer. Subgroup analysis was employed to explore the potential sources of heterogeneity. The result of subgroup analysis showed that total carotenoids (OR: 0.428; 95% CI: 0.239–0.767), α-carotene (OR: 0.640; 95% CI: 0.485–0.845), β-carotene (OR: 0.817; 95% CI: 0.709–0.942), β-cryptoxanthin (OR: 0.408; 95% CI: 0.338–0.493), and lycopene (OR: 0.674; 95% CI: 0.534–0.851) significantly decreased the risk of head and neck cancer (Table 2). The assessment of publication bias of the funnel plot by Egger’s regression test showed no publication bias in the umbrella meta-analysis (*p* = 0.83). Six imputed studies subjected to trim and fill analysis suggested that carotenoids were protective against breast cancer (OR = 0.923; 95% CI: 0.883–0.965).

### 3.10. Gynecologic/Skin/Blood Cancer Outcomes

**Figure 8:**
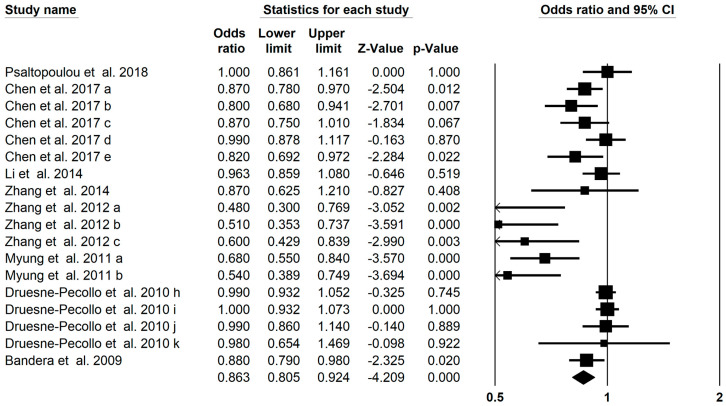
Forest plot of the effect of carotenoids on gynecologic/skin/blood cancer [28,30,34,38,43,45,56,65].

We conducted a comprehensive assessment of the limited number of meta-analyses pertaining to gynecologic/skin/blood cancers collectively, aiming to derive overall findings. The present umbrella analysis presented 18 meta-analyses of gynecologic, skin, and blood cancer studies significantly associated with carotenoids (OR = 0.928; 95% CI: 0.900–0.957; *p* < 0.001, Figure 8) with a moderate heterogeneity (I^2^ = 0.732, *p* < 0.001). Despite the paucity of available meta-analyses, we conducted separate analyses for each of the three cancers regarding total carotenoids. Further subgroup analysis showed a significant effect of total carotenoids (OR: 0.540; 95% CI: 0.433–0.672) and β-carotene (OR: 0.912; 95% CI: 0.842–0.987) on the risk of gynecologic/skin/blood cancer (Table 2). Seven meta-analyses found a significantly reduced risk of gynecologic cancer (OR: 0.683; 95% CI: 0.564–0.827). Five meta-analyses revealed insignificant reduced risk of skin cancer with carotenoids (OR: 0.991; 95% CI: 0.950–1.035). Six meta-analyses also found a significantly reduced risk of blood cancer (OR: 0.895; 95% CI: 0.832–0.962). The assessment of publication bias of the funnel plot by Egger’s regression test showed publication bias in the umbrella meta-analysis (*p* < 0.001).

### 3.11. Subgroup Analysis of Source of Carotenoids on Various Cancers

Further evaluations were conducted to detect the effects of carotenoids from different sources on various cancers. The results showed that the OR value swere not significantly changed by most of the dietary, blood, and supplement of carotenoid subgroups (Table 3). However, carotenoid supplementation significantly increased in the risk of total cancer (OR: 1.021; 95% CI: 1.000–1.043), lung cancer (OR: 1.141; 95% CI: 1.084–1.200), and bladder cancer (OR: 1.440; 95% CI: 1.000–2.090) (Table 3).

## 4. Discussion

Despite several reviews and meta-analyses evaluating the effects of carotenoids on the risk of cancer, our study aimed to provide a comprehensive overview of the available evidence. In the present umbrella meta-analysis, a total of 51 articles with 198 eligible meta-analyses were included to assess the impact of carotenoids on the most-diagnosed cancers. Total carotenoids were inversely associated with the risk of lung cancer, digestive system cancer, prostate cancer, breast cancer, head and neck cancer, gynecologic cancer, skin cancer, and blood cancer, indicating that they may have an important impact on cancer prevention, despite the presence of moderate-to-high heterogeneity among the studies.

There was sufficient evidence for a protective relationship between dietary carotenoids or serum carotenoids and cancers in the present umbrella review. The health check-up programs from 1988 to 1995 through 1998 among 3182 participants aged from 39–79 with 134 cancer deaths revealed that α-carotene, β-carotene, and lycopene reduced the risk of cancer mortality [74]. Subsequent investigations conducted on the Cancer Prevention Study II Nutrition Cohort demonstrated that serum carotenoids levels were linked to a decreased risk of breast cancer (OR: 0.86; 95% CI: 0.56–1.33; *p* = 0.74), with serum α-carotene being identified as having a significant effect on reducing the risk of breast cancer (OR: 0.50; 95% CI: 0.29–0.85; *p* = 0.041) [75]. Recently, a case-control study with 415 gastric cancer cases and 830 controls investigated the effects of dietary carotenoids on the risk of gastric cancer. The results showed that a higher intake of total dietary carotenoids and dietary lycopene was inversely associated with GC risk in women (total dietary carotenoids: OR: 0.56; 95% CI: 0.32–0.99; *p* = 0.039; dietary lycopene: OR: 0.54; 95% CI: 0.30–0.96, *p* = 0.039) [76]. The result of 11,239 prostate cancer cases and 18,541 controls from a pooled analysis of 15 studies showed lycopene significant associated with lower risk of aggressive prostate cancer (OR: 0.65; 95% CI: 0.46–0.91; *p* = 0.032), while weak evidence presented enhanced effects of α-carotene (OR: 1.06; 95% CI: 0.0.96–1.18), β-carotene (OR: 1.07; 95% CI: 0.98–1.16), zeaxanthin (OR: 1.04; 95% CI: 0.90–1.21) on prostate cancer [77]. Michaud et al. [78] found that α-carotene (OR: 0.75; 95% CI: 0.59–0.96) and lycopene (OR: 0.80; 95% CI: 0.64–0.99) intakes were significantly associated with a lower risk of lung cancer in the Nurses’ Health Study (NHS) and Health Professionals Follow-Up Study (HPFS) cohort, while the association with β-carotene, lutein, and β-cryptoxanthin intakes was inverse and non-significant. However, the conclusions of several meta-analyses are inconsistent with our results, with some reporting a significant increase in the risk of lung cancer associated with β-carotene supplementation [68,69], potentially due to cigarette smoking.

Carotenoids have been shown to possess anti-cancer properties through various mechanisms, such as inducing cell cycle arrest, promoting apoptosis, and inhibiting angiogenesis and metastasis. However, the exact effects and underlying mechanisms may vary depending on the type and stage of cancer. Previous studies have reported that carotenoids were associated with inflammation [79]. A meta-analysis study with 26 trials carried out by Fatemeh et al. [80] found that carotenoids significantly decreased C-reactive protein (CRP) (weighted mean difference (WMD): −0.54 mg/L, 95% CI: −0.71, −0.37, *p* < 0.001), and interleukin-6 (IL-6) (WMD: −0.54 pg/mL, 95% CI: −1.01, −0.06, *p* = 0.025). Moreover, lutein/zeaxanthin and β-cryptoxanthin also significantly decreased CRP level (WMD: −0.30 mg/L, 95% CI: −0.45–−0.15, *p* < 0.001; WMD: −0.35 mg/L, 95% CI: −0.54–−0.15, *p* < 0.001). In an in vitro study, Karin et al. suggested that carotenoid derivatives acted as inhibitors of the NF-κB pathway, exerting anticancer effects by inhibiting IKK kinase activity and suppressing p65 binding and transcriptional activity [81]. Furthermore, lycopene reduced the mRNA expression of inducible nitric oxide synthase and IL-6, inhibited IκB phosphorylation and degradation and NF-κB translocation, and prevented the phosphorylation of ERK1/2 and p38 MAP kinase, thus achieving an anti-inflammatory effect [82,83].

Existing evidence presented that carotenoids exhibited enhanced antioxidant properties, which is one of the potential mechanisms for preventing cancer [84]. Carotenoids scavenged radicals by donating a hydrogen atom or electron to produce a stabilized radical cation or anion that quenches reactive molecules [85]. Moreover, carotenoids can drastically reduce the risk of malignant transformation by scavenging singlet oxygen or peroxyl radical compounds, and reducing cellular damage caused by their reactions with lipids, proteins, and DNA [86]. In addition, one of the antioxidant mechanisms of carotenoids was promoting Nrf-2 localization to the nucleus, as well as promoting phase II enzyme activation to reduce oxidative stress [87]. Following radical scavenging, carotenoids enhanced the elimination of these stressed and damaged cells to prevent malignant transformation [88]. In vitro studies have demonstrated that carotenoids acted through the PI3K and MAPK pathways and induced apoptosis through PPARγ, IFNs, Bcl-2, and caspase 3/9 [89,90]. In in vivo studies, the clearance of reactive oxygen species (ROS) and promotion of cell apoptosis by multiple types of carotenoids have been found to reduce damage to organs including the liver, kidneys, and intestines [91,92,93]. However, in the Carotenoid and Retinol Efficacy Trial (CARET) [94] and the Alpha-Tocopherol Beta-Carotene Cancer Prevention Study (ATBC) [95], smokers were administered β-carotene supplements at 20 mg and 30 mg per day, which was approximately 10–20 times higher than the typical intake of an adult. The result suggested that β-carotene supplementation led to an increased mortality rate from lung cancer. One hypothesis suggests that elevated doses of carotenoids, particularly when given in isolation, may exhibit pro-oxidant activity within the lungs of smokers. A prevalent consensus existed within the scientific community, positing that a diet abundant in fruits and vegetables, distinguished by their high antioxidant content, possessed the potential to mitigate the risk of cancer. This consensus was predominantly rooted in empirical findings derived from observational studies.

However, recent fundamental research publications have introduced skepticism regarding the established notion of antioxidants’ anti-carcinogenic properties, and have cautioned that, under certain circumstances, their impact may indeed manifest as carcinogenic [96]. It was proven that high doses of a single antioxidant administered to individuals at high risk of health issues, such as smokers, were demonstrated to lack significant benefits and could potentially result in adverse effects [97]. In addition to insufficient micronutrient intake from both food and supplement sources on a daily basis, surpassing the tolerable upper intake levels is likely to present a risk of adverse health effects for nearly all individuals in the general population [98]. Henceforth, the establishment of a secure carotenoid intake necessitates the assessment of a dose–response relationship indicative of potential adverse effects on the health of animals or humans. This is also a relevant field that we aim to explore in our future research endeavors.

Our current investigation represents the initial umbrella meta-analysis to comprehensively collect and evaluate all previously published meta-analyses, culminating in a comprehensive synthesis of the available evidence pertaining to the efficacy of carotenoids in cancer prevention.

An umbrella review is the most comprehensive evaluation of previously published meta-analyses or systematic reviews, representing one of the highest levels of evidence. It also enhances the value of publications and decreases misleading outcomes, distortion, and bias. However, our study does have several limitations that need to be further considered. Firstly, we selected and included studies that were published in meta-analyses, which may have lost some studies that were not identified. Secondly, the data on total carotenoids and total cancer in the study could not be categorized. Thirdly, we only modified data that were analyzed incorrectly in the CMA and did not re-analyze all the data. Fourthly, multiple meta-analyses cited the same original observational study. Fifthly, although all studies are crowd research, including cohort studies, case-control studies, and RCT, they have different research methods and handling methods, which may affect our results. Sixthly, it was not possible to make a detailed division of intake levels, so it was not possible to verify the dose–response relationship in detail. Lastly, there is an insufficient amount of research on specific types of carotenoids in relation to various cancers, which may affect the final results. In future studies, further meta-analytical research articles are needed on the levels or ratios of carotenoid components and their associations with cancer incidence and mortality.

## 5. Conclusions

Although carotenoids are widely available in foods and commonly used as dietary supplements, and carotenoid-related studies have been published, there is no conclusive evidence regarding their protective effect on cancer risk. Our results have evaluated the most comprehensive evaluation of the relationship between carotenoids and cancer risk and found that multiple carotenoids were significantly associated with minimizing incidence and mortality of cancer. Concurrently, the findings suggest that the efficacy of carotenoid supplements in cancer prevention remains a subject of controversy, highlighting the need for cautious consideration when considering supplementation. Future study will eliminate data bias and error by analyzing individual patient data and various subgroups to likely yield more consistent results with a high level of evidence.

## Figures and Tables

**Table 1:** Summary of the meta-analyses of carotenoids and cancer risk.

Author & Year	Type of Cancer	N	Type of Studies	Type of Carotenoids	Type of Metrics	Summary Effect Size (95% CI)	Model	*I* ^2^	Egger’s *p* Value	Statistically Significant
Deng et al., 2023 a [72]	gastric cancer	3	CC, cohort	α-carotene blood	OR	0.78 (0.58, 1.05)	fixed	0.42	0.407	No
Deng et al., 2023 b [72]	gastric cancer	4	CC, cohort	β-carotene blood	OR	0.69 (0.40, 1.16)	random	0.7	0.942	No
Zhang et al., 2023 [73]	total cancer	18	RCT	β-carotene supplement	RR	1.02 (0.99, 1.05)	random	0.26	0.03	No
Yin et al., 2022 a [70]	digestive system tumors	5	RCT	β-carotene blood	OR	0.72 (0.46, 1.11)	random	0	NR	No
Yin et al., 2022 b [70]	digestive system tumors	5	RCT	lycopene blood	OR	0.93 (0.81, 1.08)	random	0	NR	No
Corbi et al., 2022 a [68]	colorectal cancer	2	RCT	β-carotene supplement	RR	0.97 (0.68, 1.38)	random	0	NR	No
Corbi et al., 2022 b [68]	esophagus and stomach cancer	2	RCT	β-carotene supplement	RR	0.93 (0.82, 1.06)	random	0	NR	No
Corbi et al., 2022 c [68]	prostate cancer	3	RCT	β-carotene supplement	RR	0.93 (0.73, 1.18)	random	0	NR	No
Corbi et al., 2022 d [68]	lung cancer	5	RCT	β-carotene supplement	RR	1.14 (1.02, 1.27)	random	0.03	NR	Yes
Corbi et al., 2022 e [68]	urinary tract cancer	2	RCT	β-carotene supplement	RR	0.82 (0.55, 1.21)	random	0	NR	No
Corbi et al., 2022 f [68]	pancreatic cancer	2	RCT	β-carotene supplement	RR	0.85 (0.62, 1.16)	random	0	NR	No
Corbi et al., 2022 g [68]	total cancer	13	RCT	β-carotene supplement	RR	0.98 (0.90, 1.07)	random	0.37	NR	No
Zhang et al., 2022 [71]	brain cancer	7	CC, cohort	β-carotene intake	RR	0.78 (0.66, 0.93)	random	0	NR	Yes
Kordiak et al., 2022 [69]	lung cancer	8	RCT	β-carotene supplement	RR	1.16 (1.06, 1.26)	fixed	0	NR	Yes
Li et al., 2020 [66]	esophageal cancer	15	CC	β-carotene intake	OR	0.62 (0.50, 0.77)	random	0.708	0.252	Yes
Wu et al., 2020 a [67]	bladder cancer	11	CC, cohort	β-carotene intake	RR	0.88 (0.76, 1.03)	random	0.748	0.07	No
Wu et al., 2020 b [67]	bladder cancer	3	CC, cohort	β-carotene blood	RR	0.36 (0.12, 1.07)	random	0.852	0.07	No
Aune et al., 2018 a [62]	total cancer	3	cohort	total carotenoid intake	RR	0.93 (0.82, 1.06)	random	0	0.42	No
Aune et al., 2018 b [62]	total cancer	5	cohort	total carotenoids blood	RR	0.74 (0.60, 0.90)	random	0	0.39	Yes
Aune et al., 2018 c [62]	total cancer	4	cohort	β-carotene intake	RR	0.90 (0.81, 1.00)	random	0	0.02	No
Aune et al., 2018 d [62]	total cancer	6	cohort	β-carotene blood	RR	0.76 (0.65, 0.89)	random	0	0.22	Yes
Aune et al., 2018 e [62]	total cancer	2	cohort	α-carotene blood	RR	0.62 (0.40, 0.96)	random	0	NR	Yes
Aune et al., 2018 f [62]	total cancer	2	cohort	β-cryptoxanthin blood	RR	0.83 (0.60, 1.15)	random	0	NR	No
Aune et al., 2018 g [62]	total cancer	3	cohort	lycopene blood	RR	0.81 (0.54, 1.21)	random	0.655	0.13	No
Psaltopoulou et al., 2018 [65]	non-Hodgkin’s lymphoma	3	CC	Lycopene intake	RR	1.00 (0.86, 1.16)	random	0	NR	No
He et al., 2018 a [64]	breast cancer mortality	5	CC, cohort	β-carotene intake	RR	0.70 (0.50, 0.99)	random	0.375	NR	Yes
He et al., 2018 b [64]	breast cancer mortality	3	CC, cohort	α-carotene intake	RR	0.97 (0.71, 1.32)	random	0.054	NR	No
He et al., 2018 c [64]	breast cancer mortality	3	CC, cohort	β-cryptoxanthin intake	RR	0.77 (0.53, 1.10)	random	0.198	NR	No
He et al., 2018 d [64]	breast cancer mortality	3	CC, cohort	lutein intake	RR	0.81 (0.42, 1.57)	random	0.769	NR	No
He et al., 2018 e [64]	breast cancer mortality	3	CC, cohort	lycopene intake	RR	0.74 (0.53, 1.03)	random	0	NR	No
Catano et al., 2018 [63]	prostate cancer	24	CC, cohort	lycopene intake	RR	0.90 (0.85, 0.95)	random	0.04	NR	Yes
Chen et al., 2017 a [56]	non-Hodgkin’s lymphoma	8	CC, cohort	α-carotene intake	RR	0.87 (0.78, 0.97)	random	0	>0.05	Yes
Chen et al., 2017 b [56]	non- Hodgkin’s lymphoma	10	CC, cohort	β-carotene intake	RR	0.80 (0.68, 0.94)	random	0.557	>0.05	Yes
Chen et al., 2017 c [56]	non- Hodgkin’s lymphoma	7	CC, cohort	β-cryptoxanthin intake	RR	0.87 (0.75, 1.01)	random	0.252	>0.05	No
Chen et al., 2017 d [56]	non- Hodgkin’s lymphoma	7	CC, cohort	lycopene intake	RR	0.99 (0.88, 1.12)	random	0	>0.05	No
Chen et al., 2017 e [56]	non- Hodgkin’s lymphoma	7	CC, cohort	lutein and zeaxanthin intake	RR	0.82 (0.69, 0.97)	random	0.448	>0.05	Yes
Panic et al., 2017 a [58]	colorectal cancer	3	CC	total carotenoid intake	OR	0.89 (0.69, 1.14)	random	0	NR	No
Panic et al., 2017 b [58]	colorectal cancer	3	CC	α-carotene intake	OR	0.58 (0.33, 1.03)	random	0.849	NR	No
Panic et al., 2017 c [58]	colorectal cancer	6	CC	β-carotene intake	OR	0.64 (0.38, 1.08)	random	0.913	NR	No
Panic et al., 2017 d [58]	colorectal cancer	2	CC	β-cryptoxanthin intake	OR	0.47 (0.12, 1.90)	random	0.965	NR	No
Panic et al., 2017 e [58]	colorectal cancer	4	CC	lycopene intake	OR	0.92 (0.46, 1.83)	random	0.947	NR	No
Panic et al., 2017 f [58]	colorectal cancer	4	CC	lutein and zeaxanthin intake	OR	0.78 (0.56, 1.09)	random	0.727	NR	No
Panic et al., 2017 g [58]	colon cancer	3	CC	β-carotene intake	OR	0.78 (0.50, 1.24)	random	0.868	NR	No
Panic et al., 2017 h [58]	colon cancer	2	CC	lycopene intake	OR	0.95 (0.79, 1.15)	random	0	NR	No
Panic et al., 2017 i [58]	colon cancer	2	CC	lutein and zeaxanthin intake	OR	0.89 (0.77, 1.03)	random	0	NR	No
Panic et al., 2017 j [58]	rectal cancer	2	CC	β-carotene intake	OR	1.13 (0.85, 1.51)	random	0	NR	No
Panic et al., 2017 k [58]	rectal cancer	2	CC	lycopene intake	OR	0.82 (0.57, 1.16)	random	0	NR	No
Panic et al., 2017 l [58]	colorectal cancer	2	cohort	total carotenoid intake	OR	1.06 (0.89, 1.27)	random	0	NR	No
Panic et al., 2017 m [58]	colorectal cancer	2	cohort	α-carotene intake	OR	1.00 (0.84, 1.18)	random	0	NR	No
Panic et al., 2017 n [58]	colorectal cancer	4	cohort	β-carotene intake	OR	0.88 (0.72, 1.07)	random	0.371	NR	No
Panic et al., 2017 o [58]	colorectal cancer	2	cohort	β-cryptoxanthin intake	OR	1.14 (0.62, 2.08)	random	0.695	NR	No
Panic et al., 2017 p [58]	colorectal cancer	3	cohort	lycopene intake	OR	0.94 (0.71, 1.24)	random	0.622	NR	No
Panic et al., 2017 q [58]	colorectal cancer	3	cohort	lutein and zeaxanthin intake	OR	0.92 (0.77, 1.09)	random	0.132	NR	No
Cui et al., 2017 [57]	prostate cancer	2	RCT	lycopene supplement	RR	0.70 (0.27, 1.85)	fixed	0.416	0.788	No
Schwingshackl et al., 2017 a [61]	total cancer mortality	3	RCT	β-carotene supplement	RR	1.12 (0.91, 1.38)	random	0.21	NR	No
Schwingshackl et al., 2017 b [61]	total cancer	2	RCT	β-carotene supplement	RR	1.09 (0.96, 1.23)	random	0.3	NR	No
Park et al., 2017 [59]	bladder cancer	3	RCT	β-carotene supplement	RR	1.44 (1.00, 2.09)	fixed	0	NR	Yes
Rowles et al., 2017 a [60]	prostate cancer	21	CC, cohort	lycopene intake	RR	0.88 (0.79, 0.99)	random	0.567	0.13	Yes
Rowles et al., 2017 b [60]	prostate cancer	17	CC, cohort	lycopene blood	RR	0.88 (0.79, 0.98)	random	0.262	0.064	Yes
Chen et al., 2016 a [51]	pancreatic cancer	3	CC, cohort	β-cryptoxanthin intake	OR	0.70 (0.56, 0.88)	random	0.284	NR	Yes
Chen et al., 2016 b [51]	pancreatic cancer	6	CC, cohort	lycopene intake	OR	0.85 (0.73, 1.00)	random	0	NR	No
Chen et al., 2016 c [51]	pancreatic cancer	4	CC, cohort	α-carotene intake	OR	0.86 (0.56, 1.33)	random	0.78	NR	No
Chen et al., 2016 d [51]	pancreatic cancer	9	CC, cohort	β-carotene intake	OR	0.74 (0.56, 0.98)	random	0.696	NR	Yes
Chen et al., 2016 e [51]	pancreatic cancer	5	CC, cohort	lutein and zeaxanthin intake	OR	0.82 (0.58, 1.15)	random	0.747	NR	No
Zhou et al., 2016 a [55]	gastric cancer	13	CC	total carotenoid intake	OR	0.62 (0.56, 0.686428571)	random	0.626	NR	Yes
Zhou et al., 2016 b [55]	gastric cancer	13	CC	β-carotene intake	OR	0.52 (0.46, 0.59)	random	0.249	NR	Yes
Zhou et al., 2016 c [55]	gastric cancer	4	CC	a-carotene intake	OR	0.58 (0.44, 0.76)	random	0.623	NR	Yes
Zhou et al., 2016 d [55]	gastric cancer	5	CC	lycopene intake	OR	0.94 (0.73, 1.21)	random	0.696	NR	No
Zhou et al., 2016 e [55]	gastric cancer	5	CC	lutein intake	OR	0.89 (0.68, 1.15)	random	0.549	NR	No
Zhou et al., 2016 f [55]	gastric cancer	8	cohort	total carotenoid intake	OR	0.82 (0.73, 0.93)	random	0.467	NR	Yes
Zhou et al., 2016 g [55]	gastric cancer	8	cohort	β-carotene intake	OR	0.74 (0.61, 0.91)	random	0.645	NR	Yes
Zhou et al., 2016 h [55]	gastric cancer	4	cohort	α-carotene intake	OR	0.79 (0.59, 1.07)	random	0.384	NR	No
Zhou et al., 2016 i [55]	gastric cancer	4	cohort	lycopene intake	OR	0.80 (0.60, 1.07)	random	0	NR	No
Zhou et al., 2016 j [55]	gastric cancer	5	cohort	lutein intake	OR	0.95 (0.77, 1.18)	random	0.454	NR	No
Abar et al., 2016 a [50]	lung cancer	7	CC, cohort	β-cryptoxanthin blood	RR	0.72 (0.45, 1.14)	random	0.69	0.23	No
Abar et al., 2016 b [50]	lung cancer	6	CC, cohort	lycopene blood	RR	0.68 (0.54, 0.87)	random	0	0	Yes
Abar et al., 2016 c [50]	lung cancer	7	CC, cohort	α-carotene blood	RR	0.70 (0.48, 1.01)	random	0.61	0.64	No
Abar et al., 2016 d [50]	lung cancer	14	CC, cohort	β-carotene blood	RR	0.71 (0.56, 0.91)	random	0.55	0.28	Yes
Abar et al., 2016 e [50]	lung cancer	6	CC, cohort	lutein and zeaxanthin blood	RR	0.86 (0.67, 1.11)	random	0	NR	No
Abar et al., 2016 f [50]	lung cancer	5	CC, cohort	total carotenoids blood	RR	0.64 (0.44, 0.93)	random	0.23	0.3	Yes
Lodi et al., 2016 [53]	oral cancer	2	RCT	β-carotene or carotenoids supplement	RR	0.71 (0.24, 2.09)	fixed	0	NR	No
Wang et al., 2016 [54]	colorectal cancer	15	CC, cohort	lycopene intake	RR	0.94 (0.80, 1.10)	random	0.805	0.864	No
Huang et al., 2016 a [52]	pancreatic cancer	23	CC, cohort	total carotenoid intake	OR	0.77 (0.67, 0.89)	random	0.569	0.17	Yes
Huang et al., 2016 b [52]	pancreatic cancer	14	CC, cohort	β-carotene intake	OR	0.78 (0.66, 0.92)	random	0.481	NR	Yes
Huang et al., 2016 c [52]	pancreatic cancer	6	CC, cohort	α-carotene intake	OR	0.88 (0.66, 1.18)	random	0.686	NR	No
Huang et al., 2016 d [52]	pancreatic cancer	7	CC, cohort	lutein and zeaxanthin intake	OR	0.80 (0.61, 1.05)	random	0.679	0.664	No
Huang et al., 2016 e [52]	pancreatic cancer	8	CC, cohort	lycopene intake	OR	0.84 (0.73, 0.97)	random	0	0.857	Yes
Huang et al., 2016 f [52]	pancreatic cancer	5	CC, cohort	β-cryptoxanthin intake	OR	0.86 (0.67, 1.12)	random	0.573	0.522	No
Yu et al., 2015 [49]	lung cancer	18	CC, cohort	β-carotene intake	RR	0.768 (0.68, 0.87)	random	0.559	0.464	Yes
Leoncini et al., 2015 a [47]	oral cavity and pharynx	2	CC	total carotenoids intake	OR	0.48 (0.19, 1.27)	random	0.933	NR	No
Leoncini et al., 2015 b [47]	larynx	1	CC	total carotenoid intake	OR	0.40 (0.19, 0.83)	random	NR	NR	Yes
Leoncini et al., 2015 c [47]	head and neck cancer	1	CC	α-carotene intake	OR	1.30 (0.66, 2.55)	random	NR	NR	No
Leoncini et al., 2015 d [47]	oral cavity and pharynx	2	CC	α-carotene intake	OR	0.57 (0.41, 0.79)	random	0	NR	Yes
Leoncini et al., 2015 e [47]	larynx	2	CC	α-carotene intake	OR	0.46 (0.20, 1.06)	random	0.831	NR	No
Leoncini et al., 2015 f [47]	head and neck cancer	1	CC	β-carotene intake	OR	1.39 (0.72, 2.67)	random	NR	NR	No
Leoncini et al., 2015 g [47]	oral cavity and pharynx	2	CC	β-carotene intake	OR	0.57 (0.14, 2.38)	random	0.939	NR	No
Leoncini et al., 2015 h [47]	epilarynx and hypopharynx	1	CC	β-carotene intake	OR	0.76 (0.47, 1.23)	random	NR	NR	No
Leoncini et al., 2015 i [47]	oral cavity	1	CC	β-carotene intake	OR	1.01 (0.68, 1.51)	random	NR	NR	No
Leoncini et al., 2015 j [47]	larynx	3	CC	β-carotene intake	OR	0.58 (0.22, 1.55)	random	0.914	NR	No
Leoncini et al., 2015 k [47]	head and neck cancer	1	CC	β-cryptoxanthin intake	OR	0.30 (0.15, 0.60)	random	NR	NR	Yes
Leoncini et al., 2015 l [47]	oral cavity and pharynx	2	CC	β-cryptoxanthin intake	OR	0.46 (0.29, 0.74)	random	0.518	NR	Yes
Leoncini et al., 2015 m [47]	larynx	2	CC	β-cryptoxanthin intake	OR	0.41 (0.33, 0.51)	random	0	NR	Yes
Leoncini et al., 2015 n [47]	head and neck cancer	1	CC	lycopene intake	OR	0.60 (0.32, 1.11)	random	NR	NR	No
Leoncini et al., 2015 o [47]	oral cavity and pharynx	4	CC	lycopene intake	OR	0.74 (0.56, 0.98)	random	0.145	NR	Yes
Leoncini et al., 2015 p [47]	larynx	4	CC	lycopene intake	OR	0.50 (0.28, 0.89)	random	0.659	NR	Yes
Leoncini et al., 2015 q [47]	head and neck cancer	1	CC	lutein and zeaxanthin intake	OR	0.95 (0.52, 1.73)	random	NR	NR	No
Leoncini et al., 2015 r [47]	oral cavity and pharynx	2	CC	lutein and zeaxanthin intake	OR	0.51 (0.22, 1.18)	random	0.83	NR	No
Leoncini et al., 2015 s [47]	larynx	2	CC	lutein and zeaxanthin intake	OR	0.60 (0.27, 1.32)	random	0.858	NR	No
Wang et al., 2015 a [48]	prostate cancer	11	CC, cohort	α-carotene blood	RR	0.91 (0.72, 1.15)	random	0.491	NR	No
Wang et al., 2015 b [48]	prostate cancer	13	CC, cohort	β-carotene blood	RR	0.96 (0.81, 1.14)	random	0.188	NR	No
Wang et al., 2015 c [48]	prostate cancer	15	CC, cohort	lycopene blood	RR	0.81 (0.69, 0.96)	random	0.233	NR	Yes
Wang et al., 2015 d [48]	prostate cancer	12	CC, cohort	α-carotene intake	RR	0.87 (0.76, 0.99)	random	0.1551	NR	Yes
Wang et al., 2015 e [48]	prostate cancer	19	CC, cohort	β-carotene intake	RR	0.90 (0.81, 1.01)	random	0.2602	NR	No
Wang et al., 2015 f [48]	prostate cancer	13	CC, cohort	lycopene intake	RR	0.88 (0.76, 1.02)	random	0.2361	NR	No
Chen et al., 2015 [46]	prostate cancer	13	CC, cohort	lycopene intake	RR	0.91 (0.82, 1.01)	random	0.455	0.22	No
Li et al., 2014 a [42]	gastric cancer	20	CC, cohort	β-carotene intake	OR	0.59 (0.49, 0.70)	random	0.687	NR	Yes
Li et al., 2014 b [42]	gastric cancer	8	CC, cohort	α-carotene intake	OR	0.69 (0.52, 0.93)	random	0.584	NR	Yes
Li et al., 2014 c [42]	gastric cancer	5	CC, cohort	β-carotene blood	OR	0.83 (0.57, 1.19)	random	0.622	NR	No
Li et al., 2014 d [42]	gastric cancer	3	CC, cohort	α-carotene blood	OR	0.79 (0.47, 1.31)	random	0.53	NR	No
Li et al., 2014 [43]	ovarian cancer	10	CC, cohort	lycopene intake	OR	0.963 (0.86, 1.08)	random	0.116	0.406	No
Tang et al., 2014 a [44]	bladder cancer	4	CC, cohort	total carotenoid intake	RR	0.67 (0.55, 0.82)	random	0	NR	Yes
Tang et al., 2014 b [44]	bladder cancer	8	CC, cohort	α-carotene intake	RR	0.87 (0.76, 0.99)	random	0.272	NR	Yes
Tang et al., 2014 c [44]	bladder cancer	12	CC, cohort	β-carotene intake	RR	0.89 (0.82, 0.97)	random	0.386	NR	Yes
Tang et al., 2014 d [44]	bladder cancer	6	CC, cohort	β-cryptoxanthin intake	RR	0.86 (0.73, 1.00)	random	0	NR	No
Tang et al., 2014 e [44]	bladder cancer	6	CC, cohort	lutein and zeaxanthin intake	RR	0.93 (0.70, 1.24)	random	0.582	NR	No
Tang et al., 2014 f [44]	bladder cancer	6	CC, cohort	lycopene intake	RR	0.95 (0.82, 1.10)	random	0	NR	No
Tang et al., 2014 g [44]	bladder cancer	2	CC, cohort	total carotenoids blood	RR	0.43 (0.20, 0.93)	random	0.273	NR	Yes
Tang et al., 2014 h [44]	bladder cancer	4	CC, cohort	α-carotene intake	RR	0.56 (0.37, 0.85)	random	0.51	NR	Yes
Tang et al., 2014 i [44]	bladder cancer	4	CC, cohort	β-carotene blood	RR	0.41 (0.05, 3.36)	random	0.724	NR	Yes
Tang et al., 2014 j [44]	bladder cancer	4	CC, cohort	β-cryptoxanthin blood	RR	0.62 (0.06, 6.41)	random	0.674	NR	No
Tang et al., 2014 k [44]	bladder cancer	4	CC, cohort	lutein and zeaxanthin blood	RR	0.50 (0.12, 0.87)	random	0.502	NR	Yes
Tang et al., 2014 l [44]	bladder cancer	4	CC, cohort	lycopene blood	RR	0.60 (0.17, 2.08)		0.61	NR	No
Zhang et al., 2014 [45]	melanoma	8	CC, cohort	β-carotene intake	OR	0.87 (0.62, 1.20)	random	0.719	0.69	No
Ge et al., 2013 a [40]	esophageal cancer	13	CC, cohort	β-carotene intake	OR	0.58 (0.44, 0.77)	random	0.782	0.114–0.962	Yes
Ge et al., 2013 b [40]	esophageal cancer	3	CC	α-carotene intake	OR	0.81 (0.70, 0.94)	fixed	0	0.114–0.962	Yes
Ge et al., 2013 c [40]	esophageal cancer	2	CC, cohort	lycopene intake	OR	0.75 (0.64, 0.88)	fixed	0	0.114–0.962	Yes
Ge et al., 2013 d [40]	esophageal cancer	3	CC, cohort	β-cryptoxanthin intake	OR	0.80 (0.66, 0.97)	random	0.509	0.114–0.962	Yes
Ge et al., 2013 e [40]	esophageal cancer	2	CC	lutein and zeaxanthin intake	OR	0.71 (0.59, 0.87)	fixed	0	0.114–0.962	Yes
Xu et al., 2013 [41]	colorectal adenoma	8	CC	lycopene intake	RR	0.87 (0.67, 1.13)	random	0.44	NR	No
Chen et al., 2013 a [39]	prostate cancer	5	CC, cohort	lycopene intake	OR	0.93 (0.86, 1.01)	random	0.18	NR	No
Chen et al., 2013 b [39]	prostate cancer	9	CC, cohort	lycopene blood	OR	0.97 (0.88, 1.07)	random	0	NR	No
Zhang et al., 2012 a [38]	cervical cancer	5	CC	total carotenoids blood	OR	0.48 (0.30, 0.77)	random	0.69	NR	Yes
Zhang et al., 2012 b [38]	cervical cancer	8	CC	total carotenoid intake	OR	0.51 (0.35, 0.73)	random	0.82	NR	Yes
Zhang et al., 2012 c [38]	cervical cancer	3	CC	total carotenoid intake	OR	0.60 (0.43, 0.84)	random	0.51	NR	Yes
Hu et al., 2012 a [37]	breast cancer	10	CC	α-carotene intake	OR	0.82 (0.70, 0.97)	random	0.6632	0.3	Yes
Hu et al., 2012 b [37]	breast cancer	25	CC	β-carotene intake	OR	0.76 (0.67, 0.86)	random	0.6767	0.01	Yes
Hu et al., 2012 c [37]	breast cancer	6	cohort	α-carotene intake	OR	0.91 (0.85, 0.98)	random	0	0.54	Yes
Hu et al., 2012 d [37]	breast cancer	10	cohort	β-carotene intake	OR	0.95 (0.90, 1.00)	random	0	0.48	No
Aune et al., 2012 a [35]	breast cancer	3	CC, cohort	total carotenoid intake	RR	0.95 (0.84, 1.08)	random	0.66	NR	No
Aune et al., 2012 b [35]	breast cancer	7	CC, cohort	total carotenoid blood	RR	0.74 (0.57, 0.96)	random	0.53	NR	Yes
Aune et al., 2012 c [35]	breast cancer	10	CC, cohort	β-carotene intake	RR	0.93 (0.88, 0.98)	random	0	NR	Yes
Aune et al., 2012 d [35]	breast cancer	14	CC, cohort	β-carotene blood	RR	0.82 (0.64, 1.04)	random	0.55	NR	No
Aune et al., 2012 e [35]	breast cancer	2	CC, cohort	β-carotene supplement	RR	1.08 (0.96, 1.22)	random	0	NR	No
Aune et al., 2012 f [35]	breast cancer	6	CC, cohort	α-carotene intake	RR	0.93 (0.86, 1.01)	random	0.16	NR	No
Aune et al., 2012 g [35]	breast cancer	12	CC, cohort	α-carotene blood	RR	0.80 (0.68, 0.95)	random	0.15	NR	Yes
Aune et al., 2012 h [35]	breast cancer	6	CC, cohort	β-cryptoxanthin intake	RR	1.02 (0.95, 1.09)	random	0	NR	No
Aune et al., 2012 i [35]	breast cancer	10	CC, cohort	β-cryptoxanthin blood	RR	0.89 (0.76, 1.05)	random	0	NR	No
Jeon et al., 2011 a [33]	total cancer	6	RCT	β-carotene supplement	RR	1.08 (0.99, 1.18)	random	0.54	0.41	No
Jeon et al., 2011 b [33]	total cancer mortality	4	RCT	β-carotene supplement	RR	1.00 (0.87, 1.15)	fixed	0	0.41	No
Myung et al., 2011 a [34]	cervical neoplasm	9	CC	β-carotene intake	OR	0.68 (0.55, 0.84)	fixed	0.321	NR	Yes
Myung et al., 2011 b [34]	cervical neoplasm	5	CC	lycopene intake	OR	0.54 (0.39, 0.75)	fixed	0.044	NR	Yes
Ilic et al., 2011 [32]	prostate cancer	3	RCT	lycopene supplement	RR	0.67 (0.36, 1.23)	random	0	0.859	No
Druesne-Pecollo et al., 2010 a [30]	total cancer	8	RCT	β-carotene supplement	RR	1.01 (0.98, 1.04)	fixed	NR	NR	No
Druesne-Pecollo et al., 2010 b [30]	lung cancer	8	RCT	β-carotene supplement	RR	1.13 (1.04, 1.23)	fixed	NR	NR	Yes
Druesne-Pecollo et al., 2010 c [30]	stomach cancer	7	RCT	β-carotene supplement	RR	0.99 (0.86, 1.13)	fixed	NR	NR	No
Druesne-Pecollo et al., 2010 d [30]	pancreas cancer	4	RCT	β-carotene supplement	RR	0.99 (0.73, 1.36)	fixed	NR	NR	No
Druesne-Pecollo et al., 2010 e [30]	colon-rectum cancer	7	RCT	β-carotene supplement	RR	0.96 (0.85, 1.09)	fixed	NR	NR	No
Druesne-Pecollo et al., 2010 f [30]	prostate cancer	5	RCT	β-carotene supplement	RR	0.99 (0.91, 1.07)	fixed	NR	NR	No
Druesne-Pecollo et al., 2010 g [30]	breast cancer	4	RCT	β-carotene supplement	RR	0.96 (0.85, 1.08)	fixed	NR	NR	No
Druesne-Pecollo et al., 2010 h [30]	non melanoma	4	RCT	β-carotene supplement	RR	0.99 (0.93, 1.05)	fixed	NR	NR	No
Druesne-Pecollo et al., 2010 i [30]	basal cells cancer	3	RCT	β-carotene supplement	RR	1.00 (0.93, 1.07)	fixed	NR	NR	No
Druesne-Pecollo et al., 2010 j [30]	squamous cells cancer	3	RCT	β-carotene supplement	RR	0.99 (0.86, 1.14)	fixed	NR	NR	No
Druesne-Pecollo et al., 2010 k [30]	melanoma	3	RCT	β-carotene supplement	RR	0.98 (0.65, 1.46)	fixed	NR	NR	No
Jiang et al., 2010 [31]	prostate cancer	3	RCT	β-carotene supplement	RR	0.97 (0.90, 1.05)	random	0	NR	No
Veloso et al., 2009 a [29]	total cancer	11	cohort	β-carotene intake/blood	OR/RR	1.01 (0.88, 1.16)	NR	NR	NR	No
Veloso et al., 2009 b [29]	total cancer	9	cohort	lycopene intake/blood	OR/RR	0.99 (0.94, 1.05)	NR	NR	NR	No
Veloso et al., 2009 c [29]	total cancer	7	cohort	α-carotene intake/blood	OR/RR	0.91 (0.78, 1.05)	NR	NR	NR	No
Veloso et al., 2009 d [29]	total cancer	7	cohort	β-cryptoxanthin intake/blood	OR/RR	1.08 (0.95, 1.23)	NR	NR	NR	No
Veloso et al., 2009 e [29]	total cancer	17	Nested CC	β-carotene intake/blood	OR/RR	0.98 (0.86, 1.11)	NR	NR	NR	No
Veloso et al., 2009 f [29]	total cancer	14	Nested CC	lycopene intake/blood	OR/RR	0.87 (0.77, 0.99)	NR	NR	NR	Yes
Veloso et al., 2009 g [29]	total cancer	14	Nested CC	α-carotene intake/blood	OR/RR	0.96 (0.79, 1.17)	NR	NR	NR	No
Veloso et al., 2009 h [29]	total cancer	17	Nested CC	β-cryptoxanthin intake/blood	OR/RR	0.94 (0.83, 1.07)	NR	NR	NR	No
Veloso et al., 2009 i [29]	total cancer	29	CC	β-carotene intake/blood	OR/RR	0.73 (0.64, 0.83)	NR	NR	NR	Yes
Veloso et al., 2009 j [29]	total cancer	24	CC	lycopene intake/blood	OR/RR	0.76 (0.64, 0.91)	NR	NR	NR	Yes
Veloso et al., 2009 k [29]	total cancer	20	CC	α-carotene intake/blood	OR/RR	0.75 (0.64, 0.88)	NR	NR	NR	Yes
Veloso et al., 2009 l [29]	total cancer	20	CC	β-cryptoxanthin intake/blood	OR/RR	0.74 (0.63, 0.88)	NR	NR	NR	Yes
Bandera et al., 2009 [28]	endometrial cancer	8	CC, cohort	β-carotene intake	OR	0.88 (0.79, 0.98)	random	0.777	NR	Yes
Gallicchio et al., 2008 a [26]	lung cancer	8	cohort	total carotenoids intake	RR	0.79 (0.71, 0.88)	random	0	NR	Yes
Gallicchio et al., 2008 b [26]	lung cancer	11	cohort	β-carotene intake	RR	0.92 (0.83, 1.02)	random	0	NR	No
Gallicchio et al., 2008 c [26]	lung cancer	6	RCT	β-carotene supplement	RR	1.10 (0.89, 1.36)	random	NR	NR	No
Gallicchio et al., 2008 d [26]	lung cancer	4	cohort	total carotenoids serum	RR	0.70 (0.44, 1.11)	random	0.46	NR	No
Gallicchio et al., 2008 e [26]	lung cancer	10	cohort	β-carotene serum	RR	0.84 (0.66, 1.07)	random	0	NR	No
Tanvetyanon et al., 2008 [27]	lung cancer	4	CC, cohort	β-carotene intake	OR	1.21 (1.09, 1.34)	random	0.325	NR	Yes
Bjelakovic et al., 2006 [25]	colorectal adenoma	4	RCT	β-carotene supplement	RR	0.93 (0.67, 1.30)	random	0.651	NR	No
Bjelakovic et al., 2004 [23]	gastrointestinal cancers	5	RCT	β-carotene supplement	RR	0.99 (0.85, 1.15)	fixed	0.173	NR	No
Etminan et al., 2004 a [24]	prostate cancer	10	CC, cohort	lycopene intake	RR	0.89 (0.81, 0.98)	random	NR	NR	Yes
Etminan et al., 2004 b [24]	prostate cancer	7	CC, cohort	lycopene blood	RR	0.74 (0.59, 0.92)	random	NR	NR	Yes
Gandini et al., 2000 [22]	breast cancer	11	CC, cohort	β-carotene intake	RR	0.82 (0.76, 0.88)	random	NR	NR	Yes

N, number of meta-analyses; RCT, randomized controlled trial; CC, case control; CI, confidence interval; OR, odds ratio; RR, relative risk; NR, not reported.

**Table 2:** Subgroup analysis of types of carotenoids on various cancers.

Type of Cancer	Type of Carotenoids	No. of Meta-Analyses	OR (95% CI)	*I*^2^ (*p* Value)
Total cancer	total carotenoids	19	0.743 (0.675–0.819)	0.748 (<0.001)
	α-carotene	28	0.838 (0.797–0.881)	0.416 (0.012)
	β-carotene	77	0.906 (0.875–0.938)	0.816 (<0.001)
	lutein and zeaxanthin	16	0.850 (0.797–0.906)	0 (<0.001)
	β-cryptoxanthin	19	0.785 (0.697–0.883)	0.826 (<0.001)
	lycopene	39	0.886 (0.858–0.916)	0.391 (0.008)
Lung cancer	total carotenoids	3	0.774 (0.700–0.855)	0 (0.518)
	α-carotene	1	0.700 (0.480–1.010)	NA (NA)
	β-carotene	9	0.998 (0.892–1.117)	0.866 (<0.001)
	lutein and zeaxanthin	1	0.860 (0.670–1.110)	NA (NA)
	β-cryptoxanthin	1	0.720 (0.450–1.140)	NA (NA)
	lycopene	1	0.680 (0.540–0.870)	NA (NA)
Digestive system cancer	total carotenoids	5	0.811 (0.674–0.975)	0.875 (<0.001)
	α-carotene	10	0.792 (0.707–0.887)	0.384 (0.102)
	β-carotene	22	0.799 (0.717–0.890)	0.810 (<0.001)
	lutein and zeaxanthin	8	0.856 (0.794–0.923)	0 (0.528)
	β-cryptoxanthin	5	0.790 (0.698–0.894)	0 (0.479)
	lycopene	12	0.873 (0.825–0.924)	0 (0.770)
gastric cancer	total carotenoids	17	0.749 (0.668–0.841)	0.806 (<0.001)
colorectal cancer	total carotenoids	22	0.932 (0.887–0.979)	0 (0.867)
esophageal cancer	total carotenoids	7	0.752 (0.671–0.844)	0.653 (0.008)
pancreas cancer	total carotenoids	13	0.812 (0.765–0.861)	0 (0.935)
Prostate cancer	total carotenoids	NA	NA	NA (NA)
	α-carotene	2	0.880 (0.784–0.987)	0 (0.743)
	β-carotene	5	0.961 (0.917–1.007)	0 (0.735)
	lutein and zeaxanthin	NA	NA	NA (NA)
	β-cryptoxanthin	NA	NA	NA (NA)
	lycopene	12	0.899 (0.872–0.927)	0 (0.612)
Breast cancer	total carotenoids	2	0.862 (0.678–1.094)	0.651 (0.091)
	α-carotene	5	0.900 (0.857–0.945)	0.027 (0.391)
	β-carotene	8	0.896 (0.833–0.964)	0.764 (<0.001)
	lutein and zeaxanthin	1	0.810 (0.420–1.570)	NA (NA)
	β-cryptoxanthin	3	0.944 (0.824–1.081)	0.525 (0.122)
	lycopene	1	0.740 (0.530–1.030)	NA (NA)
Bladder cancer	total carotenoids	2	0.631 (0.469–0.849)	0.167 (0.273)
	α-carotene	2	0.731 (0.479–1.115)	0.746 (0.047)
	β-carotene	5	0.931 (0.774–1.120)	0.585 (0.047)
	lutein and zeaxanthin	2	0.908 (0.687–1.200)	0 (0.403)
	β-cryptoxanthin	2	0.859 (0.734–1.005)	0 (0.784)
	lycopene	2	0.944 (0.816–1.093)	0 (0.478)
Head and neck cancer	total carotenoids	2	0.428 (0.239–0.767)	0 (0.766)
	α-carotene	3	0.640 (0.485–0.845)	0.623 (0.070)
	β-carotene	7	0.817 (0.709–0.942)	0 (0.574)
	lutein and zeaxanthin	3	0.719 (0.474–1.090)	0 (0.434)
	β-cryptoxanthin	3	0.408 (0.338–0.493)	0 (0.604)
	lycopene	3	0.674 (0.534–0.851)	0 (0.452)
Gynecologic/skin/blood cancer	total carotenoids	3	0.540 (0.433–0.672)	0 (0.700)
	α-carotene	1	0.870 (0.780–0.970)	NA (NA)
	β-carotene	8	0.912 (0.842–0.987)	0.655 (0.005)
	lutein and zeaxanthin	1	0.820 (0.690–0.970)	NA (NA)
	β-cryptoxanthin	1	0.870 (0.750–1.010)	NA (NA)
	lycopene	4	0.905 (0.773–1.058)	0.758 (0.006)
gynecologic cancer	total carotenoids	7	0.683 (0.564–0.827)	0.819 (<0.001)
skin cancer	total carotenoids	5	0.991 (0.950–1.035)	0 (0.956)
blood cancer	total carotenoids	6	0.859 (0.832–0.962)	0.379 (0.154)

CI, confidence interval; OR, odds ratio; NA, not available.

**Table 3:** Subgroup analysis of source of carotenoids on various cancers.

Type of Cancer	Source of Carotenoids	No. of Meta-Analyses	OR (95% CI)	*I*^2^ (*p* Value)
Total cancer	Carotenoids intake	118	0.823 (0.797–0.849)	0.740 (<0.001)
	Carotenoids serum	32	0.807 (0.765–0.851)	0.278 (0.075)
	Carotenoids supplement	32	1.021 (1.000–1.043)	0.227 (0.126)
Lung cancer	Carotenoids intake	4	0.908 (0.739–1.116)	0.929 (<0.001)
	Carotenoids serum	8	0.744 (0.670–0.826)	0 (0.810)
	Carotenoids supplement	4	1.141 (1.084–1.200)	0 (0.959)
Digestive system cancer	Carotenoids intake	48	0.798 (0.754–0.844)	0.683 (<0.001)
	Carotenoids serum	6	0.864 (0.773–0.967)	0 (0.705)
	Carotenoids supplement	8	0.960 (0.902–1.021)	0 (0.990)
Prostate cancer	Carotenoids intake	8	0.900 (0.871–0.930)	0 (0.990)
	Carotenoids serum	6	0.892 (0.827–0.962)	0.337 (0.183)
	Carotenoids supplement	4	0.974 (0.923–1.028)	0 (0.629)
Breast cancer	Carotenoids intake	9	0.906 (0.860–0.954)	0.734 (<0.001)
	Carotenoids serum	4	0.826 (0.749–0.910)	0 (0.649)
	Carotenoids supplement	2	1.019 (0.908–1.143)	0.453 (0.176)
Head and neck cancer	Carotenoids intake	20	0.634 (0.530–0.758)	0.617 (<0.001)
	Carotenoids serum	1	0.710 (0.240–2.090)	NA (NA)
	Carotenoids supplement	NA	NA	NA (NA)
Bladder cancer	Carotenoids intake	8	0.854 (0.789–0.923)	0.460 (0.073)
	Carotenoids serum	6	0.451 (0.274–0.741)	0 (0.993)
	Carotenoids supplement	1	1.440 (1.000–2.090)	NA (NA)
Gynecologic/skin/blood cancer	Carotenoids intake	13	0.829 (0.762–0.902)	0.688 (<0.001)
	Carotenoids serum	1	0.480 (0.300–0.770)	NA (NA)
	Carotenoids supplement	4	0.994 (0.952–1.038)	0 (0.997)

CI, confidence interval; OR, odds ratio; NA, not available.

## Data Availability

The original contributions presented in the study are included in the article/Supplementary Material, further inquiries can be directed to the corresponding author.

## Supplementary Materials

The following supporting information can be downloaded at: https://www.mdpi.com/article/10.3390/foods13091321/s1, Figure S1: Forest plot of the effect of carotenoids on total cancer.; Table S1 Results of risk of bias assessment based on AMSTAR 2 tool.
