# Supplementary material for: The Efficacy of Dietary Intake, Supplementation, and Blood Concentrations of Carotenoids in Cancer Prevention: Insights from an Umbrella Meta-Analysis
Source: Foods. 2024 Apr 25;13(9):1321. doi: 10.3390/foods13091321 (PMC11083701; doi:10.3390/foods13091321)
Supplement: Supplementary file 1 [file foods-13-01321-s001.zip › foods-2977226-supplementary.pdf]

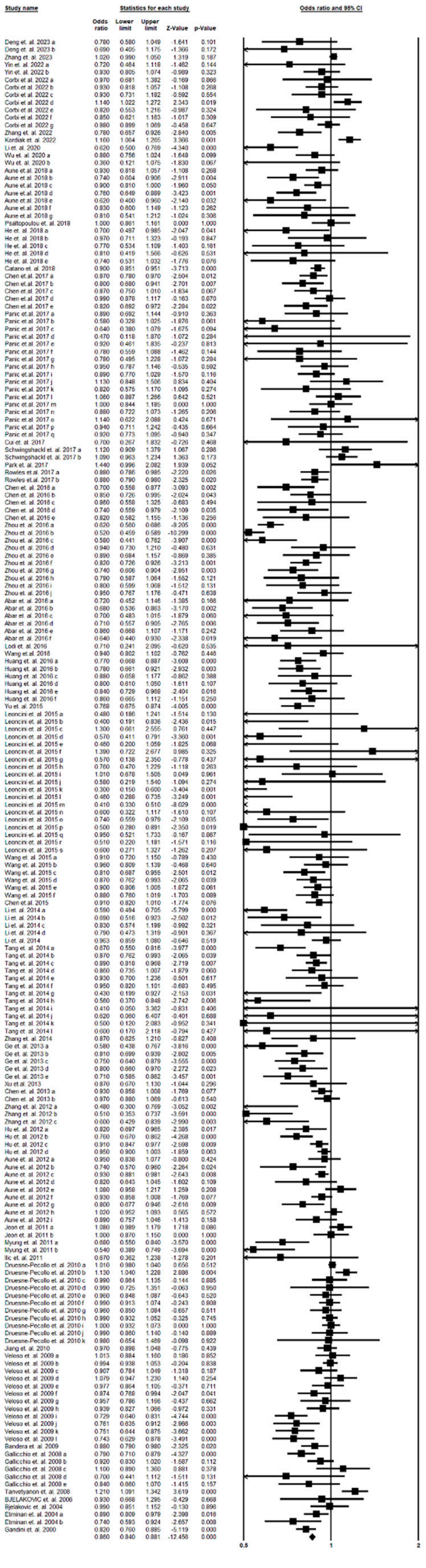

Figure S1 Forest plot of the effect of carotenoids on total cancer.

Table S1 Results of risk of bias assessment based on AMSTAR 2 tool

Author & year	Q1	Q2	Q3	Q4	Q5	Q6	Q7	Q8	Q9	Q10	Q11	Q12	Q13	Q14	Q15	Q16	OC
Deng et al. 2023 a	Y	Y	Y	Y	Y	Y	Y	Y	Y	Y	Y	N	N	N	N	Y	CL
Deng et al. 2023 b	Y	Y	Y	Y	Y	Y	Y	Y	Y	Y	Y	N	N	N	N	Y	CL
Zhang et al. 2023	Y	Y	Y	Y	N	Y	Y	Y	Y	Y	Y	Y	Y	N	Y	Y	L
Yin et al. 2022 a	N	Y	Y	Y	N	N	N	Y	Y	Y	Y	N	N	N	N	Y	CL
Yin et al. 2022 b	N	Y	Y	Y	N	N	N	Y	Y	Y	Y	N	N	N	N	Y	CL
Corbi et al. 2022 a	Y	Y	Y	Y	Y	Y	Y	Y	Y	Y	Y	Y	Y	Y	Y	Y	H
Corbi et al. 2022 b	Y	Y	Y	Y	Y	Y	Y	Y	Y	Y	Y	Y	Y	Y	Y	Y	H
Corbi et al. 2022 c	Y	Y	Y	Y	Y	Y	Y	Y	Y	Y	Y	Y	Y	Y	Y	Y	H
Corbi et al. 2022 d	Y	Y	Y	Y	Y	Y	Y	Y	Y	Y	Y	Y	Y	Y	Y	Y	H
Corbi et al. 2022 e	Y	Y	Y	Y	Y	Y	Y	Y	Y	Y	Y	Y	Y	Y	Y	Y	H
Corbi et al. 2022 f	Y	Y	Y	Y	Y	Y	Y	Y	Y	Y	Y	Y	Y	Y	Y	Y	H
Corbi et al. 2022 g	Y	Y	Y	Y	Y	Y	Y	Y	Y	Y	Y	Y	Y	Y	Y	Y	H
Zhang et al. 2022	Y	Y	Y	Y	N	Y	Y	Y	Y	Y	Y	Y	Y	Y	Y	Y	M
Kordiak et al. 2022	Y	N	Y	Y	Y	Y	Y	Y	N	Y	Y	N	N	Y	N	Y	CL
Li et al. 2020	Y	N	Y	Y	N	Y	Y	Y	Y	N	Y	Y	Y	Y	Y	N	L
Wu et al. 2020 a	Y	Y	Y	Y	N	Y	Y	Y	Y	Y	Y	Y	Y	Y	Y	Y	H
Wu et al. 2020 b	Y	Y	Y	Y	N	Y	Y	Y	Y	Y	Y	Y	Y	Y	Y	Y	H
Aune et.al. 2018 a	Y	N	Y	Y	N	Y	Y	Y	Y	Y	Y	Y	Y	Y	Y	Y	L
Aune et.al. 2018 b	Y	N	Y	Y	N	Y	Y	Y	Y	Y	Y	Y	Y	Y	Y	Y	L
Aune et.al. 2018 c	Y	N	Y	Y	N	Y	Y	Y	Y	Y	Y	Y	Y	Y	Y	Y	L
Aune et.al. 2018 d	Y	N	Y	Y	N	Y	Y	Y	Y	Y	Y	Y	Y	Y	Y	Y	L
Aune et.al. 2018 e	Y	N	Y	Y	N	Y	Y	Y	Y	Y	Y	Y	Y	Y	Y	Y	L
Aune et.al. 2018 f	Y	N	Y	Y	N	Y	Y	Y	Y	Y	Y	Y	Y	Y	Y	Y	L
Aune et.al. 2018 g	Y	N	Y	Y	N	Y	Y	Y	Y	Y	Y	Y	Y	N	Y	Y	L
Psaltopoulou et al. 2018	Y	Y	Y	N	N	Y	Y	Y	Y	Y	Y	Y	Y	Y	Y	Y	L
He et al. 2018 a	Y	Y	Y	Y	Y	Y	Y	Y	Y	N	Y	Y	Y	Y	Y	Y	H
He et al. 2018 b	Y	Y	Y	Y	Y	Y	Y	Y	Y	N	Y	Y	Y	Y	Y	Y	H
He et al. 2018 c	Y	Y	Y	Y	Y	Y	Y	Y	Y	N	Y	Y	Y	Y	Y	Y	H
He et al. 2018 d	Y	Y	Y	Y	Y	Y	Y	Y	Y	N	Y	Y	Y	Y	Y	Y	H
He et al. 2018 e	Y	Y	Y	Y	Y	Y	Y	Y	Y	N	Y	Y	Y	Y	Y	Y	H
Catano et al. 2018	Y	Y	Y	Y	Y	Y	Y	Y	Y	Y	Y	N	N	Y	N	Y	CL
Chen et.al. 2017 a	Y	Y	Y	Y	N	Y	Y	Y	Y	Y	Y	Y	Y	Y	Y	Y	H
Chen et.al. 2017 b	Y	Y	Y	Y	N	Y	Y	Y	Y	Y	Y	Y	Y	N	Y	Y	M
Chen et.al. 2017 c	Y	Y	Y	Y	N	Y	Y	Y	Y	Y	Y	Y	Y	Y	Y	Y	H
Chen et.al. 2017 d	Y	Y	Y	Y	N	Y	Y	Y	Y	Y	Y	Y	Y	Y	Y	Y	H
Chen et.al. 2017 e	Y	Y	Y	Y	N	Y	Y	Y	Y	Y	Y	Y	Y	Y	Y	Y	H
Panic et.al. 2017 a	Y	N	N	Y	N	Y	Y	Y	N	Y	Y	N	N	Y	Y	Y	CL
Panic et.al. 2017 b	Y	N	N	Y	N	Y	Y	Y	N	Y	Y	N	N	Y	Y	Y	CL
Panic et.al. 2017 c	Y	N	N	Y	N	Y	Y	Y	N	Y	Y	N	N	Y	Y	Y	CL
Panic et.al. 2017 d	Y	N	N	Y	N	Y	Y	Y	N	Y	Y	N	N	Y	Y	Y	CL
Panic et.al. 2017 e	Y	N	N	Y	N	Y	Y	Y	N	Y	Y	N	N	Y	Y	Y	CL
Panic et.al. 2017 f	Y	N	N	Y	N	Y	Y	Y	N	Y	Y	N	N	Y	Y	Y	CL
Panic et.al. 2017 g	Y	N	N	Y	N	Y	Y	Y	N	Y	Y	N	N	Y	Y	Y	CL
Panic et.al. 2017 h	Y	N	N	Y	N	Y	Y	Y	N	Y	Y	N	N	Y	Y	Y	CL
Panic et.al. 2017 i	Y	N	N	Y	N	Y	Y	Y	N	Y	Y	N	N	Y	Y	Y	CL
Panic et.al. 2017 j	Y	N	N	Y	N	Y	Y	Y	N	Y	Y	N	N	Y	Y	Y	CL
Panic et.al. 2017 k	Y	N	N	Y	N	Y	Y	Y	N	Y	Y	N	N	Y	Y	Y	CL
Panic et.al. 2017 l	Y	N	N	Y	N	Y	Y	Y	N	Y	Y	N	N	Y	Y	Y	CL
Panic et.al. 2017 m	Y	N	N	Y	N	Y	Y	Y	N	Y	Y	N	N	Y	Y	Y	CL
Panic et.al. 2017 n	Y	N	N	Y	N	Y	Y	Y	N	Y	Y	N	N	Y	Y	Y	CL

Panic et.al. 2017 o	Y	N	N	Y	N	Y	Y	Y	N	Y	Y	N	N	Y	Y	Y	CL
Panic et.al. 2017 p	Y	N	N	Y	N	Y	Y	Y	N	Y	Y	N	N	Y	Y	Y	CL
Panic et.al. 2017 q	Y	N	N	Y	N	Y	Y	Y	N	Y	Y	N	N	Y	Y	Y	CL
Cui et al. 2017	Y	Y	Y	Y	N	N	Y	Y	Y	Y	Y	Y	Y	Y	Y	Y	M
Schwingshackl et al. 2017 a	Y	Y	Y	Y	Y	Y	Y	Y	Y	Y	Y	N	N	N	N	Y	CL
Schwingshackl et al. 2017 b	Y	Y	Y	Y	Y	Y	Y	Y	Y	Y	Y	N	N	N	N	Y	CL
Park et al. 2017	Y	Y	Y	Y	N	Y	Y	Y	Y	N	Y	Y	Y	Y	Y	Y	M
Rowles et.al. 2017 a	Y	Y	Y	Y	Y	N	Y	Y	Y	N	Y	Y	N	N	Y	Y	M
Rowles et.al. 2017 b	Y	Y	Y	Y	Y	N	Y	Y	Y	N	Y	Y	N	Y	Y	Y	M
Chen et al. 2016 a	Y	Y	Y	Y	N	Y	Y	Y	Y	Y	Y	N	N	Y	N	Y	CL
Chen et al. 2016 b	Y	Y	Y	Y	N	Y	Y	Y	Y	Y	Y	N	N	Y	N	Y	CL
Chen et al. 2016 c	Y	Y	Y	Y	N	Y	Y	Y	Y	Y	Y	N	N	Y	N	Y	CL
Chen et al. 2016 d	Y	Y	Y	Y	N	Y	Y	Y	Y	Y	Y	N	N	Y	N	Y	CL
Chen et al. 2016 e	Y	Y	Y	Y	N	Y	Y	Y	Y	Y	Y	N	N	Y	N	Y	CL
Zhou et al. 2016 a	Y	Y	Y	Y	N	Y	Y	Y	Y	Y	Y	Y	Y	N	Y	Y	M
Zhou et al. 2016 b	Y	Y	Y	Y	N	Y	Y	Y	Y	Y	Y	Y	Y	Y	Y	Y	H
Zhou et al. 2016 c	Y	Y	Y	Y	N	Y	Y	Y	Y	Y	Y	Y	Y	N	Y	Y	M
Zhou et al. 2016 d	Y	Y	Y	Y	N	Y	Y	Y	Y	Y	Y	Y	Y	N	Y	Y	M
Zhou et al. 2016 e	Y	Y	Y	Y	N	Y	Y	Y	Y	Y	Y	Y	Y	N	Y	Y	M
Zhou et al. 2016 f	Y	Y	Y	Y	N	Y	Y	Y	Y	Y	Y	Y	Y	Y	Y	Y	H
Zhou et al. 2016 g	Y	Y	Y	Y	N	Y	Y	Y	Y	Y	Y	Y	Y	N	Y	Y	M
Zhou et al. 2016 h	Y	Y	Y	Y	N	Y	Y	Y	Y	Y	Y	Y	Y	Y	Y	Y	H
Zhou et al. 2016 i	Y	Y	Y	Y	N	Y	Y	Y	Y	Y	Y	Y	Y	Y	Y	Y	H
Zhou et al. 2016 j	Y	Y	Y	Y	N	Y	Y	Y	Y	Y	Y	Y	Y	Y	Y	Y	H
Abar et al. 2016 a	Y	Y	Y	N	N	N	Y	Y	Y	Y	Y	Y	Y	Y	Y	Y	L
Abar et al. 2016 b	Y	Y	Y	N	N	N	Y	Y	Y	Y	Y	Y	Y	Y	Y	Y	L
Abar et al. 2016 c	Y	Y	Y	N	N	N	Y	Y	Y	Y	Y	Y	Y	Y	Y	Y	L
Abar et al. 2016 d	Y	Y	Y	N	N	N	Y	Y	Y	Y	Y	Y	Y	Y	Y	Y	L
Abar et al. 2016 e	Y	Y	Y	N	N	N	Y	Y	Y	Y	Y	Y	Y	Y	Y	Y	L
Abar et al. 2016 f	Y	Y	Y	N	N	N	Y	Y	Y	Y	Y	Y	Y	Y	Y	Y	L
Lodi et al. 2016	Y	Y	N	N	N	N	Y	Y	Y	Y	Y	Y	Y	Y	Y	Y	L
Wang et.al. 2016	Y	Y	N	Y	N	N	Y	Y	Y	N	Y	Y	Y	Y	Y	N	M
Huang et al. 2016 a	Y	Y	Y	Y	N	Y	Y	Y	Y	Y	Y	Y	Y	Y	Y	Y	H
Huang et al. 2016 b	Y	Y	Y	Y	N	Y	Y	Y	Y	Y	Y	Y	Y	Y	N	Y	H
Huang et al. 2016 c	Y	Y	Y	Y	N	Y	Y	Y	Y	Y	Y	Y	Y	Y	Y	Y	H
Huang et al. 2016 d	Y	Y	Y	Y	N	Y	Y	Y	Y	Y	Y	Y	Y	Y	Y	Y	H
Huang et al. 2016 e	Y	Y	Y	Y	N	Y	Y	Y	Y	Y	Y	Y	Y	Y	Y	Y	H
Huang et al. 2016 f	Y	Y	Y	Y	N	Y	Y	Y	Y	Y	Y	Y	Y	Y	Y	Y	H
Yu et al. 2015	Y	Y	Y	Y	N	N	Y	Y	Y	N	Y	Y	Y	Y	Y	Y	M
Leoncini et al. 2015 a	Y	Y	Y	Y	N	Y	Y	Y	N	Y	Y	N	N	Y	N	Y	CL
Leoncini et al. 2015 b	Y	Y	Y	Y	N	Y	Y	Y	N	Y	Y	N	N	Y	N	Y	CL
Leoncini et al. 2015 c	Y	Y	Y	Y	N	Y	Y	Y	N	Y	Y	N	N	Y	N	Y	CL
Leoncini et al. 2015 d	Y	Y	Y	Y	N	Y	Y	Y	N	Y	Y	N	N	Y	N	Y	CL
Leoncini et al. 2015 e	Y	Y	Y	Y	N	Y	Y	Y	N	Y	Y	N	N	Y	N	Y	CL
Leoncini et al. 2015 f	Y	Y	Y	Y	N	Y	Y	Y	N	Y	Y	N	N	Y	N	Y	CL
Leoncini et al. 2015 g	Y	Y	Y	Y	N	Y	Y	Y	N	Y	Y	N	N	Y	N	Y	CL
Leoncini et al. 2015 h	Y	Y	Y	Y	N	Y	Y	Y	N	Y	Y	N	N	Y	N	Y	CL
Leoncini et al. 2015 i	Y	Y	Y	Y	N	Y	Y	Y	N	Y	Y	N	N	Y	N	Y	CL
Leoncini et al. 2015 j	Y	Y	Y	Y	N	Y	Y	Y	N	Y	Y	N	N	Y	N	Y	CL
Leoncini et al. 2015 k	Y	Y	Y	Y	N	Y	Y	Y	N	Y	Y	N	N	Y	N	Y	CL
Leoncini et al. 2015 l	Y	Y	Y	Y	N	Y	Y	Y	N	Y	Y	N	N	Y	N	Y	CL

Leoncini et al. 2015 m	Y	Y	Y	Y	N	Y	Y	Y	N	Y	Y	N	N	Y	N	Y	CL
Leoncini et al. 2015 n	Y	Y	Y	Y	N	Y	Y	Y	N	Y	Y	N	N	Y	N	Y	CL
Leoncini et al. 2015 o	Y	Y	Y	Y	N	Y	Y	Y	N	Y	Y	N	N	Y	N	Y	CL
Leoncini et al. 2015 p	Y	Y	Y	Y	N	Y	Y	Y	N	Y	Y	N	N	Y	N	Y	CL
Leoncini et al. 2015 q	Y	Y	Y	Y	N	Y	Y	Y	N	Y	Y	N	N	Y	N	Y	CL
Leoncini et al. 2015 r	Y	Y	Y	Y	N	Y	Y	Y	N	Y	Y	N	N	Y	N	Y	CL
Leoncini et al. 2015 s	Y	Y	Y	Y	N	Y	Y	Y	N	Y	Y	N	N	Y	N	Y	CL
Wang et al. 2015 a	Y	Y	Y	Y	N	Y	Y	Y	N	Y	Y	N	N	N	N	Y	CL
Wang et al. 2015 b	Y	Y	Y	Y	N	Y	Y	Y	N	Y	Y	N	N	N	N	Y	CL
Wang et al. 2015 c	Y	Y	Y	Y	N	Y	Y	Y	N	Y	Y	N	N	N	N	Y	CL
Wang et al. 2015 d	Y	Y	Y	Y	N	Y	Y	Y	N	Y	Y	N	N	N	N	Y	CL
Wang et al. 2015 e	Y	Y	Y	Y	N	Y	Y	Y	N	Y	Y	N	N	N	N	Y	CL
Wang et al. 2015 f	Y	Y	Y	Y	N	Y	Y	Y	N	Y	Y	N	N	N	N	Y	CL
Chen et.al. 2015	Y	Y	Y	Y	Y	Y	Y	Y	Y	Y	Y	Y	Y	Y	Y	Y	H
Li et al. 2014 a	Y	Y	Y	Y	N	Y	Y	Y	N	Y	Y	N	N	N	N	Y	CL
Li et al. 2014 b	Y	Y	Y	Y	N	Y	Y	Y	N	Y	Y	N	N	N	N	Y	CL
Li et al. 2014 c	Y	Y	Y	Y	N	Y	Y	Y	N	Y	Y	N	N	N	N	Y	CL
Li et al. 2014 d	Y	Y	Y	Y	N	Y	Y	Y	N	Y	Y	N	N	N	N	Y	CL
Li et al. 2014	Y	Y	Y	Y	N	Y	Y	Y	Y	Y	Y	Y	Y	Y	Y	Y	H
Tang et al. 2014 a	Y	Y	Y	Y	N	Y	Y	Y	N	N	Y	N	N	Y	N	Y	CL
Tang et al. 2014 b	Y	Y	Y	Y	N	Y	Y	Y	N	N	Y	N	N	Y	N	Y	CL
Tang et al. 2014 c	Y	Y	Y	Y	N	Y	Y	Y	N	N	Y	N	N	Y	N	Y	CL
Tang et al. 2014 d	Y	Y	Y	Y	N	Y	Y	Y	N	N	Y	N	N	Y	N	Y	CL
Tang et al. 2014 e	Y	Y	Y	Y	N	Y	Y	Y	N	N	Y	N	N	N	N	Y	CL
Tang et al. 2014 f	Y	Y	Y	Y	N	Y	Y	Y	N	N	Y	N	N	Y	N	Y	CL
Tang et al. 2014 g	Y	Y	Y	Y	N	Y	Y	Y	N	N	Y	N	N	Y	N	Y	CL
Tang et al. 2014 h	Y	Y	Y	Y	N	Y	Y	Y	N	N	Y	N	N	N	N	Y	CL
Tang et al. 2014 i	Y	Y	Y	Y	N	Y	Y	Y	N	N	Y	N	N	N	N	Y	CL
Tang et al. 2014 j	Y	Y	Y	Y	N	Y	Y	Y	N	N	Y	N	N	N	N	Y	CL
Tang et al. 2014 k	Y	Y	Y	Y	N	Y	Y	Y	N	N	Y	N	N	N	N	Y	CL
Tang et al. 2014 l	Y	Y	Y	Y	N	Y	Y	Y	N	N	Y	N	N	N	N	Y	CL
Zhang et al. 2014	Y	Y	Y	Y	N	Y	Y	Y	Y	Y	Y	Y	Y	N	Y	Y	M
Ge et al. 2013 a	Y	Y	Y	Y	Y	Y	Y	Y	Y	Y	Y	Y	Y	Y	Y	Y	H
Ge et al. 2013 b	Y	Y	Y	Y	Y	Y	Y	Y	Y	Y	Y	Y	Y	N	Y	Y	H
Ge et al. 2013 c	Y	Y	Y	Y	Y	Y	Y	Y	Y	Y	Y	Y	Y	Y	Y	Y	H
Ge et al. 2013 d	Y	Y	Y	Y	Y	Y	Y	Y	Y	Y	Y	Y	Y	Y	Y	Y	H
Ge et al. 2013 e	Y	Y	Y	Y	Y	Y	Y	Y	Y	Y	Y	Y	Y	N	Y	Y	H
Xu et.al. 2013	Y	Y	Y	Y	Y	Y	Y	Y	N	N	Y	N	N	Y	N	Y	CL
Chen et al. 2013 a	Y	Y	Y	Y	N	Y	Y	Y	Y	Y	Y	Y	Y	Y	Y	Y	H
Chen et al. 2013 b	Y	Y	Y	Y	N	Y	Y	Y	Y	Y	Y	Y	Y	Y	Y	Y	H
Zhang et al. 2012 a	Y	Y	Y	Y	N	Y	Y	Y	Y	Y	Y	N	N	Y	N	Y	CL
Zhang et al. 2012 b	Y	Y	Y	Y	N	Y	Y	Y	Y	Y	Y	N	N	Y	N	Y	CL
Zhang et al. 2012 c	Y	Y	Y	Y	N	Y	Y	Y	Y	Y	Y	N	N	Y	N	Y	CL
Hu et al. 2012 a	Y	Y	Y	Y	N	Y	Y	Y	Y	N	Y	Y	Y	Y	Y	Y	M
Hu et al. 2012 b	Y	Y	Y	Y	N	Y	Y	Y	Y	N	Y	Y	N	Y	N	Y	CL
Hu et al. 2012 c	Y	Y	Y	Y	N	Y	Y	Y	Y	N	Y	Y	Y	Y	Y	Y	M
Hu et al. 2012 d	Y	Y	Y	Y	N	Y	Y	Y	Y	N	Y	Y	Y	Y	Y	Y	M
Aune et al. 2012 a	Y	Y	Y	Y	N	Y	Y	Y	Y	Y	Y	N	N	Y	N	Y	CL
Aune et al. 2012 b	Y	Y	Y	Y	N	Y	Y	Y	Y	Y	Y	N	N	Y	N	Y	CL
Aune et al. 2012 c	Y	Y	Y	Y	N	Y	Y	Y	Y	Y	Y	N	N	Y	N	Y	CL
Aune et al. 2012 d	Y	Y	Y	Y	N	Y	Y	Y	Y	Y	Y	N	N	Y	N	Y	CL
Aune et al. 2012 e	Y	Y	Y	Y	N	Y	Y	Y	Y	Y	Y	N	N	Y	N	Y	CL
Aune et al. 2012 f	Y	Y	Y	Y	N	Y	Y	Y	Y	Y	Y	N	N	Y	N	Y	CL

Aune et al. 2012 g	Y	Y	Y	Y	N	Y	Y	Y	Y	Y	Y	N	N	Y	N	Y	CL
Aune et al. 2012 h	Y	Y	Y	Y	N	Y	Y	Y	Y	Y	Y	N	N	Y	N	Y	CL
Aune et al. 2012 i	Y	Y	Y	Y	N	Y	Y	Y	Y	Y	Y	N	N	Y	N	Y	CL
Jeon et al. 2011 a	Y	Y	Y	Y	Y	Y	Y	Y	Y	Y	Y	Y	Y	N	Y	Y	H
Jeon et al. 2011 b	Y	Y	Y	Y	Y	Y	Y	Y	Y	Y	Y	Y	Y	Y	Y	Y	H
Myung et al. 2011 a	Y	Y	Y	Y	N	Y	Y	Y	N	Y	Y	N	N	Y	N	Y	CL
Myung et al. 2011 b	Y	Y	Y	Y	N	Y	Y	Y	N	Y	Y	N	N	Y	N	Y	CL
Ilic et al. 2011	Y	Y	Y	Y	Y	Y	Y	Y	Y	Y	Y	Y	Y	Y	Y	Y	H
Druesne-Pecollo et al. 2010 a	Y	Y	Y	Y	Y	Y	Y	Y	N	Y	Y	N	N	N	N	Y	CL
Druesne-Pecollo et al. 2010 b	Y	Y	Y	Y	Y	Y	Y	Y	N	Y	Y	N	N	N	N	Y	CL
Druesne-Pecollo et al. 2010 c	Y	Y	Y	Y	Y	Y	Y	Y	N	Y	Y	N	N	N	N	Y	CL
Druesne-Pecollo et al. 2010 d	Y	Y	Y	Y	Y	Y	Y	Y	N	Y	Y	N	N	N	N	Y	CL
Druesne-Pecollo et al. 2010 e	Y	Y	Y	Y	Y	Y	Y	Y	N	Y	Y	N	N	N	N	Y	CL
Druesne-Pecollo et al. 2010 f	Y	Y	Y	Y	Y	Y	Y	Y	N	Y	Y	N	N	N	N	Y	CL
Druesne-Pecollo et al. 2010 g	Y	N	Y	Y	Y	Y	Y	Y	N	Y	Y	N	N	N	N	Y	CL
Druesne-Pecollo et al. 2010 h	Y	N	Y	Y	Y	Y	Y	Y	N	Y	Y	N	N	N	N	Y	CL
Druesne-Pecollo et al. 2010 i	Y	Y	Y	Y	Y	Y	Y	Y	N	Y	Y	N	N	N	N	Y	CL
Druesne-Pecollo et al. 2010 j	Y	N	Y	Y	Y	Y	Y	Y	N	Y	Y	N	N	N	N	Y	CL
Druesne-Pecollo et al. 2010 k	Y	Y	Y	Y	Y	Y	Y	Y	N	Y	Y	N	N	N	N	Y	CL
Jiang et al. 2010	Y	N	Y	Y	N	Y	Y	Y	N	Y	Y	N	N	Y	N	Y	CL
Veloso et al. 2009 a	Y	Y	Y	Y	N	Y	Y	Y	N	N	Y	N	N	N	N	N	CL
Veloso et al. 2009 b	Y	Y	Y	Y	N	Y	Y	Y	N	N	Y	N	N	N	N	N	CL
Veloso et al. 2009 c	Y	Y	Y	Y	N	Y	Y	Y	N	N	Y	N	N	N	N	N	CL
Veloso et al. 2009 d	Y	Y	Y	Y	N	Y	Y	Y	N	N	Y	N	N	N	N	N	CL
Veloso et al. 2009 e	Y	Y	Y	Y	N	Y	Y	Y	N	N	Y	N	N	N	N	N	CL
Veloso et al. 2009 f	Y	Y	Y	Y	N	Y	Y	Y	N	N	Y	N	N	N	N	N	CL
Veloso et al. 2009 g	Y	Y	Y	Y	N	Y	Y	Y	N	N	Y	N	N	N	N	N	CL
Veloso et al. 2009 h	Y	Y	Y	Y	N	Y	Y	Y	N	N	Y	N	N	N	N	N	CL
Veloso et al. 2009 i	Y	Y	Y	Y	N	Y	Y	Y	N	N	Y	N	N	N	N	N	CL
Veloso et al. 2009 j	Y	Y	Y	Y	N	Y	Y	Y	N	N	Y	N	N	N	N	N	CL
Veloso et al. 2009 k	Y	Y	Y	Y	N	Y	Y	Y	N	N	Y	N	N	N	N	N	CL
Veloso et al. 2009 l	Y	Y	Y	Y	N	Y	Y	Y	N	N	Y	N	N	N	N	N	CL
Bandera et al. 2009	Y	Y	Y	Y	Y	Y	Y	Y	Y	Y	Y	N	N	Y	N	Y	CL
Gallicchio et al. 2008 a	Y	Y	Y	Y	N	Y	Y	Y	Y	Y	Y	N	N	Y	N	Y	CL
Gallicchio et al. 2008 b	Y	Y	Y	Y	N	Y	Y	Y	Y	Y	Y	N	N	Y	N	Y	CL
Gallicchio et al. 2008 c	Y	Y	Y	Y	N	Y	Y	Y	Y	Y	Y	N	N	N	N	Y	CL
Gallicchio et al. 2008 d	Y	Y	Y	Y	N	Y	Y	Y	Y	Y	Y	N	N	N	N	Y	CL
Gallicchio et al. 2008 e	Y	Y	Y	Y	N	Y	Y	Y	Y	Y	Y	N	N	Y	N	Y	CL
Tanvetyanon et al. 2008	Y	Y	Y	Y	N	N	Y	Y	N	Y	Y	Y	N	Y	N	Y	CL
BJELAKOVIC et al. 2006	Y	Y	Y	Y	N	N	Y	Y	Y	Y	Y	N	N	Y	N	Y	CL
Bjelakovic et al. 2004	Y	Y	Y	Y	N	N	Y	Y	N	Y	Y	N	N	Y	N	Y	CL

Etminan et al. 2004 a	Y	Y	Y	Y	N	N	Y	Y	Y	Y	Y	Y	Y	N	Y	Y	M
Etminan et al. 2004 b	Y	Y	Y	Y	N	N	Y	Y	Y	Y	Y	Y	Y	N	Y	Y	M
Gandini et al. 2000	Y	Y	Y	N	N	N	Y	Y	Y	Y	Y	Y	N	Y	N	Y	CL

AMSTAR 2: a Measurement Tool to Assess the Methodological Quality of Systematic Reviews. AMSTAR 2 evaluation items (the items in bold are considered critical): Q1: Did the research questions and inclusion criteria for the review include the components of PICO? Q2: Did the report of the review contain an explicit statement that the review methods were established prior to the conduct of the review and did the report justify any significant deviations from the protocol? Q3: Did the review authors explain their selection of the study designs for inclusion in the review? Q4: Did the review authors use a comprehensive literature search strategy? Q5: Did the review authors perform study selection in duplicate? Q6: Did the review authors perform data extraction in duplicate? Q7: Did the review authors provide a list of excluded studies and justify the exclusions? Q8: Did the review authors describe the included studies in adequate detail? Q9: Did the review authors use a satisfactory technique for assessing the risk of bias in individual studies that were included in the review? Q10: Did the review authors report on the sources of funding for the studies included in the review? Q11: If meta-analysis was performed, did the review authors use appropriate methods for statistical combination of results? Q12: If meta-analysis was performed, did the review authors assess the potential impact of risk of bias in individual studies on the results of the meta-analysis or other evidence synthesis? Q13: Did the review authors account for risk of bias in primary studies when interpreting/discussing the results of the review? Q14: Did the review authors provide a satisfactory explanation for, and discussion of, any heterogeneity observed in the results of the review? Q15: If they performed quantitative synthesis did the review authors carry out an adequate investigation of publication bias (small study bias) and discuss its likely impact on the results of the review? Q16: Did the review authors report any potential sources of conflict of interest, including any funding they received for conducting the review?
OC: Overall confidence; H: High; M: Moderate; L: Low; CL: Critically low; Y:Yes; N: No.
